# Single-cell Transcriptome Profiling reveals Dermal and Epithelial cell fate decisions during Embryonic Hair Follicle Development

**DOI:** 10.7150/thno.44306

**Published:** 2020-06-12

**Authors:** Wei Ge, Shao-Jing Tan, Shan-He Wang, Lan Li, Xiao-Feng Sun, Wei Shen, Xin Wang

**Affiliations:** 1 Key Laboratory of Animal Genetics, Breeding and Reproduction of Shaanxi Province, College of Animal Science and Technology, Northwest A&F University, Yangling, Shaanxi 712100, China.; 2 College of Life Sciences, Qingdao Agricultural University, Qingdao 266109, China.

**Keywords:** Single-cell transcriptome, Cell fate decision, Hair follicle morphogenesis

## Abstract

It is estimated that 50% of men and 25% of women worldwide suffer from hair loss, and therefore it is of great significance to investigate the molecular pathways driving hair follicle de novo morphogenesis. However, due to high cellular heterogeneity and the asynchronous development of hair follicles, our current understanding of the molecular mechanisms involved in follicle development remains limited.

**Methods:** Single-cell suspensions from the dorsal skin of E13.5 (induction stage), E16.5 (organogenesis) fetal mice, and newborn mice (cytodifferentiation stage, postnatal day 0, P0) were prepared for unbiased single-cell RNA sequencing. To delineate the single-cell transcriptional landscape during hair follicle de novo morphogenesis, we performed t-distributed Stochastic Neighbor Embedding (tSNE), pseudotime cell trajectory inference, and regulon enrichment analysis to dissect cellular heterogeneity and reveal the molecular pathways underlying major cell type cell fate decisions. To validate our analysis, we further performed immunohistochemistry analysis of the key molecules involved during hair follicle morphogenesis. Meanwhile, intercellular communication between different cell populations was inferred based on a priori knowledge of ligand-receptor pairs.

**Results:** Based on tSNE analysis, we identified 14 cell clusters from skin tissue and delineated their cellular identity from specific gene expression profiles. By using pseudotime ordering analysis, we successfully constructed the epithelium/dermal cell lineage differentiation trajectory. For dermal cell lineage, our analysis here recapitulated the dynamic gene expression profiles during dermal condensate (DC) cell fate commitment and delineated the heterogeneity of the different dermal papilla (DP) cell populations during in utero hair follicle development. For the epithelium cell lineage, our analysis revealed the dynamic gene expression profiles of the underappreciated matrix, interfollicular epidermis (IFE), hair shaft and inner root sheath (IRS) cell populations. Furthermore, single-cell regulatory network inference and clustering analysis revealed key regulons during cell fate decisions. Finally, intercellular communication analysis demonstrated that strong intercellular communication was involved during early hair follicle development.

**Conclusions:** Our findings here provide a molecular landscape during hair follicle epithelium/dermal cell lineage fate decisions, and recapitulate the sequential activation of core regulatory transcriptional factors (TFs) in different cell populations during hair follicle morphogenesis. More importantly, our study here represents a valuable resource for understanding the molecular pathways involved during hair follicle de novo morphogenesis, which will have implications for future hair loss treatments.

## Introduction

It is estimated that 50% of men and 25% of women worldwide suffer from hair loss, and currently, transplantation of autologous hair follicles has become the main treatment strategy for this hair loss 1, 2. However, for patients without adequate autologous hair follicles, the transplantation of heterologous hair follicles remains ineffective 3. To address this problem, induction of hair follicles from autologous derived stem cells has become a hotspot for research efforts. However, our current understanding on hair follicle de novo morphogenesis *in vivo* remains limited due to the high heterogeneity and the asynchronous development of hair follicles 4, 5. From this perspective, revealing the molecular pathways underlying hair follicle de novo morphogenesis will provide in-depth insights into hair follicle development and will have implications for the induction of hair follicle development under *in vitro* conditions.

In mice, *in utero* hair follicle development has been histologically categorized into three unique stages: induction (E13.5 - E14.5), organogenesis (E15.5 - 17.5), and cytodifferentiation (E18.5 onwards) 5. More recently, with the development of single-cell RNA sequencing (scRNA-seq), new intermediate cell states during early hair follicle morphogenesis have been delineated and an updated classification of different hair follicle stages has been reported 6, 7. Seminal works have delineated that reciprocal signaling pathways between the epithelial and dermal cell populations play vital roles during hair follicle morphogenesis 8-11. However, our current knowledge regarding *in utero* hair follicle morphogenesis remains limited.

At ~E13.5 in mice, the unspecified epidermis receives signals from the mesenchyme (also known as “first dermal signal”) and subsequently forms a layer of thickened epithelial known as placodes. This marks the earliest morphological characteristic during the initiation of hair follicle morphogenesis 12, 13. Wnt/β-Catenin and Eda/Edar/NF-κB signaling have been demonstrated to play vital roles during placode fate commitment 14, 15, while the upstream regulators remain elusive. Following placode fate commitment, they signal to the underlying fibroblasts to promote the formation of DC, the precursor of the DP. The signal/s involved in the “first epithelial signal” remain largely unknown. However, fibroblast growth factor 20 (Fgf20) signaling has been shown to be one of the “first epithelial signals” as ablation of Fgf20 in mice results in the failure of DC formation 16. After the commitment of the placode and DC, the cross talk then promotes the transition to the next stage of development: signals from DC, also known as the “second dermal signal”, promote the downward proliferation of epithelial placode cells and whereafter, it's believed that Wnt and Shh signaling to promote these epithelial cells to encircle the DP in the dermal layer 8, 17, 18. Interestingly, it has been demonstrated that the further development of the epidermal is independent of hair follicle signaling and the suprabasal cells arise at ~E13.5 and gradually give rise to the IFE 19. After the envelopment of the DC by epithelial cells, the DC then matures into the DP surrounded with matrix cell populations. As the cross-talk between the DP and surrounding matrix continues, signals from the DP then promote the surrounding matrix cells to further differentiate into the hair shaft and IRS. At this time, the rudiment of a developing hair follicle becomes morphologically evident.

While the process of hair follicle morphogenesis has been well-documented, our current understanding of the molecular signatures and gene regulatory networks operating within a particular cell population remains limited. Also, limited progress has been made to identify conserved gene markers expressed in the different cell populations. By using genetic loss-of-function assays, transgenic mouse models, and flow cytometer cell sorting technology based on prior knowledge of well-defined markers, the molecular signatures of different cell populations during hair follicle development have been reported 7, 20, 21. However, the molecular signatures are varied resulting in groups using different cell markers to identify particular cell populations 21.

It is also worth noting that during early hair follicle development the molecular signature of a particular cell population may change dramatically with the intermediate cellular states remaining to be elucidated 7. Another confounding issue is that hair follicle development is asynchronous: guard hair follicles are induced as early as ~E13.5, and awl/auchene hair follicles are formed at ~E15.5, while the zigzag hair follicle, which make up about 80 % of adult hairs, initiates morphogenesis at ~E17.5 22, 23. Our current understanding of the timing and the machinery underlying the growth of different hair follicles remains limited. Driskell *et al*., demonstrated that *Sox2* expression in the DC may participate in controlling the hair follicle type, as evidenced by the fact that from E18.5 SOX2 expression is confined to a subset of cells (guard/awl/auchene dermal papillae, G/AA-DP, but not zigzag dermal papillae, ZZ-DP). This provides evidence that DP cells in different types of hair follicles are heterogeneous 20. Supporting such a notion, *Sox18* ablation in mice results in the loss of zigzag hairs 24, 25. Conversely, Chi *et al*., demonstrated that the number of DP cells in the hair follicle correlates with the hair follicle type during development, suggesting that the different number of DPs induced different cumulative signaling, which then specifies the hair size and type 26.

Tackling these problems using scRNA-seq, two recent back-to-back studies have uncovered new intermediate states that form during DC specification 7, 27. This has helped fill in the gaps regarding the underappreciated intermediate cellular states occurring during cell fate determination. It also has provided new molecular insights into the cellular heterogeneity within particular cell populations, during hair follicle development. Here, also by utilizing the same scRNA-seq technology, we performed integrated analysis on 15,086 single cell transcriptomes from E13.5 and the underappreciated E16.5 and P0 mouse dorsal skin which encompasses hair follicle induction, organogenesis, and the cytodifferentiation stages, respectively. By using tSNE analysis we identified 9 major cell populations. Based on Monocle pseudotime ordering analysis and the SCENIC regulon inferring assay we successfully constructed the epithelium/dermal cell lineage differentiation trajectory and revealed sequential activation of key regulons involved during cell fate decisions. The intercellular communications occurring were also inferred during hair follicle morphogenesis. Taken together, our data provide new insights into cell fate decisions occurring during hair follicle *in uterus* development, and more importantly, delineates molecular information regarding the underappreciated organogenesis, and cytodifferentiation stages.

## Results

### Single-cell sequencing and characterization of cellular heterogeneity during hair follicle morphogenesis

**Figure 1:**
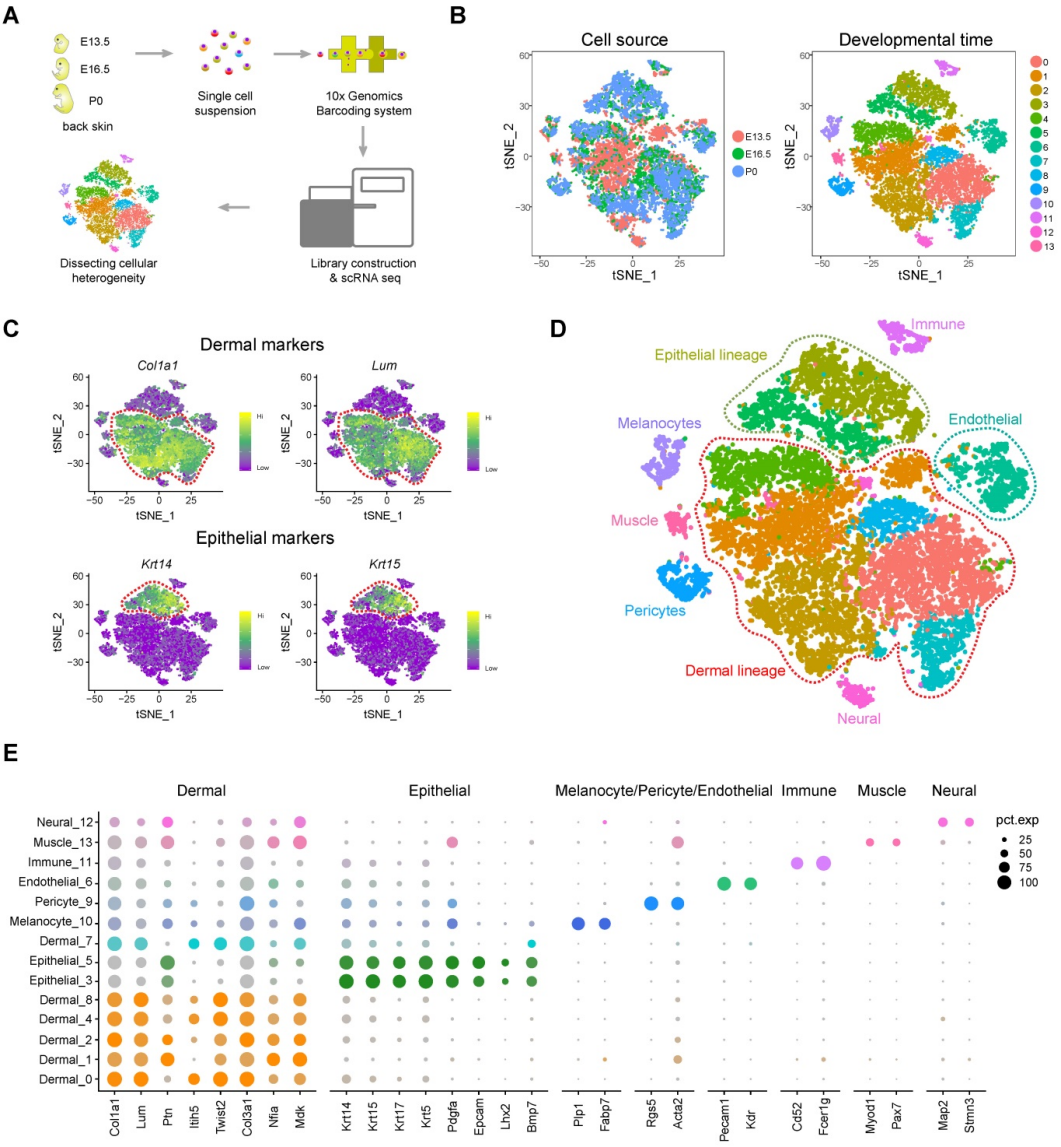
** Overview of experimental procedures and characterization of major cell populations from embryonic skin tissues.** (A) Schematic diagram illustrating the experimental pipeline for scRNA-seq analysis of embryonic skin tissues. Single-cell transcriptomes were obtained based on the 10x Chromium platform. (B) tSNE plot of embryonic single skin cells. Each point represents one single cell and cells in the same cluster represents high similarity in transcriptome profile. The left plot depicts tSNE plot of the integrated dataset from 3 different time point and cells were color-coded with developmental time point. The right plot depicts 14 transcriptional distinct cell clusters and cells were color-coded with cluster information. (C) Visualization of dermal and epithelial marker gene expression across all single cells in the tSNE plot.* Col1a1* and *Lum* were used to mark dermal cell populations and *Krt14* and *Krt15* were used to mark epithelium populations. (D) Characterization of major cell types in the embryonic skin tissues in the tSNE plot. Cells were labeled with their cell identity and were color-coded. (E) Dot plot depicts representative dermal cell, epithelial, melanocyte, and pericyte gene expression. The dot size represents the percentage of cells expressed and the color intensity represents relative expression level.

To decipher the transcriptome regulatory network and cellular fate decisions during hair follicle morphogenesis, we dissociated dorsal back skin tissue from three timepoints during hair follicle development, the induction stage (E13.5), organogenesis stage (E16.5), and the cytodifferentiation stage (P0), into single cells and performed droplet-based scRNA-seq (Figure 1A). We detected 19,997 genes in total for E13.5 skin cells, 19,767 genes for E16.5 skin cells and 19,145 genes for P0 skin cells (Figure S1A-B). After removing low-quality cells, we obtained 15,086 single-cell transcriptome profiles from E13.5, E16.5 and P0 mouse back skin cells (4,994, 5,152, and 4,940 single cells, respectively).

To dissect the cellular heterogeneity during hair follicle morphogenesis, we then performed tSNE analysis of all the single cells (Figure 1B). We found that only a small percentage of the cells from E13.5 overlapped with cells from E16.5 and P0 (Figure 1B, left panel and Figure S1C). Furthermore, tSNE analysis revealed 14 cell clusters according to their gene expression profiles and most of the clusters consisted of cells from different developmental time-points (Figure S1D), thus preliminarily deciphering that skin cells were highly heterogenous (Figure 1B, right panel). To further characterize cell cluster identity we initially performed hierarchical clustering on the 14 cell clusters (Figure S2A) and the results revealed 8 major branches (cluster 12; cluster 11; cluster 3, 5; cluster 10; cluster 1, 4, 8, 13; cluster 0, 2, 7; cluster 6; cluster 9). We then evaluated the expression of a series of well-recognized cell marker genes and revealed 8 major cell identities: *Col1a1* and *Lum* highly expressing dermal cell clusters (Figure 1C, top panel) 27, 28; *Krt14* and *Krt15* highly expressing epithelial cell cluster (Figure 1C, lower panel) 29; *Pecam1* and *Kdr* highly expressing endothelial cell cluster 30; *Plp1* and *Fabp7* highly expressing melanocyte cell cluster 31; *Rgs5* and *Acta2* highly expressing pericyte cell cluster 32; *Map2* and *Stmn3* highly expressing neural cell cluster 33; *Myod1* and *Pax7* highly expressing muscle cell cluster 34, and *Cd52* and *Fcer1g* highly expressing immune cell cluster (Figure S2B) 35. These results further validated our hierarchical clustering analysis. Together, these analyses here enable *bona fide* characterization of different cell populations within the skin tissues (Figure 1D). Besides, we also used a recently published Seurat V3 pipeline to validate our tSNE analysis, and the result also consistent with our tSNE analysis (Figure S2C).

Based on Seurat analysis, we also compared the cluster-specific gene expression across the cell clusters (Figure 1E) and it was found that dermal cell clusters showed high expression levels of *Col1a1*, *Lum*, *Ptn*, *Twist2*, *Col3a1*, *Nfia* and *Mdk*, while epithelial cells showed high expression of *Krt14*, *Krt15*, *Krt17*, *Krt5*, *Pdgfa* and *Bmp7*. We also revealed DC cell cluster specific genes including, *Sox18*, *Sox2*, *Trps1*, *Foxd1*, *Bmp3* and *Cpne5*. Visualization of the top 10 expressed cluster specific genes showed obvious cluster specific expression (Figure S2D and Table S1). Taken together, we have successfully identified major cell populations in the dorsal skin during hair follicle morphogenesis and identified a series of cell identity specific signature genes, which enable* bona fide* characterization of cellular heterogeneity.

### Revealing dermal cell fate decisions during hair follicle induction stage (E13.5 - E16.5)

**Figure 2:**
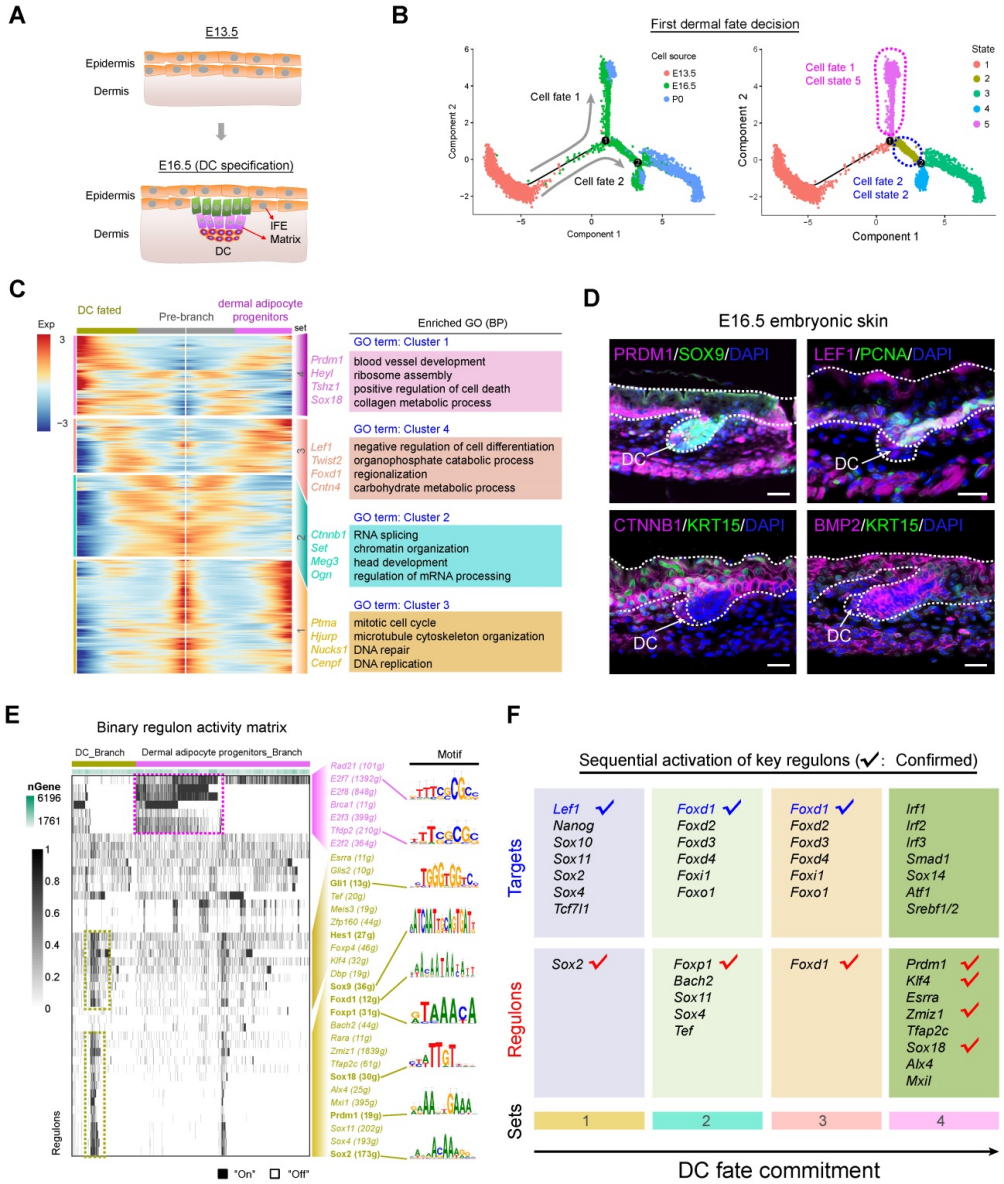
** Recapitulating dermal cell fate decision towards DC fate.** (A) Diagram deciphering dermal lineage and epidermal lineage differentiation at E13.5 to E16.5. (B) Pseudotime ordering of all dermal cell lineage cells. Each dot represents one cell and each branch represents one cell state. The left plot was labeled with developmental time and the right plot was labeled with cell states. (C) Heatmap illustrating the (differentially expressed genes, DEGs) dynamics towards DC and fibroblast fate along pseudotime. The DEGs were clustered into 4 gene sets according to *k*-means and the expression curve was illustrated in the middle. GO terms enriched for each gene set were labeled in the right panel. DC fated represents cell fate 2 in Figure 2B, while dermal adipocyte progenitors represent cell fate 1 in Figure 2B. (D) Immunofluorescence analysis of PRDM1, SOX9, LEF1, PCNA, CTNNB1, KRT15 and BMP2 expression in the E16.5 dorsal skin. Scale bars, 50 μm. (E) SCENIC binary regulon activity heatmap depicting DC and fibroblast enriched regulons. The column depicts a single cell while the row depicts regulons. For the regulons of particular interest, their representative binding motif was visualized in the right panel. “On” depicts active, while “Off” represents inactive. (F) Sequential visualization of enriched regulon activity in each gene set corresponding to Figure 2C. Their representative target genes were also provided and the red ticks depict confirmed markers in DC fate commitment.

From E13.5 to E16.5, unspecified dermal and epidermal cells differentiate into DC and IFE or matrix precursors, respectively (Figure 2A). We initially focused on the dermal cells and extracted all dermal lineage cells (dermal cell clusters and DC cluster in Figure 1D) and performed pseudotime ordering based on the Monocle algorithm (Figure 2B and Figure S3A). After pseudotime analysis, we observed two branches across the hair follicle morphogenesis stage. The first branch point 1 mainly consists of cells from E16.5, while branch point 2 consists of cells from P0 (Figure 2B), thus deciphering major cell fate decisions during dermal cell differentiation across the hair follicle *in utero* differentiation. To further verify the directionality in the pseudotime trajectory, we performed RNA velocity analysis to estimate the expression of unspliced and spliced mRNAs in the trajectory 36. The RNA velocity analysis also showed a clear directional flow at the end of each branch point, further corroborating the Monocle analysis (Figure S3B). However, it's also worth noting that some RNA velocity vectors do not follow specific trajectories, which may be caused by the discontinuous sampling method and asynchronous development of hair follicles.

We next focused on the branch point 1 and performed gene expression analysis along each branch (Figure 2C). According to the pseudotime ordering analysis, dermal cells bifurcated into two cellular states (state 2 and state 5 in Figure 2B, right panel). To reveal the sequential gene expression dynamics along each branch we visualized the gene expression dynamics along the pseudotime trajectory (Table S2) and observed four distinct gene sets according to their expression pattern (Figure 2C and Figure S3C). For the pre-branch (gene set 1), namely unspecified dermal cells from E13.5, they showed high-level expression of *Ptma*, *Hjurp*, *Nucks1*, *Cenpf*, and enriched GO terms of “mitotic cell cycle, microtubule cytoskeleton organization, and DNA repair”, which was similar to the dermal adipocyte progenitors (cell fate 1). While for the DC fated branch (cell fate 2) we found three subsequent stages (gene set 2, 3, 4). For gene set 2 we observed elevated expression of *Igfbp5*, *Set*, *Meg3* and these genes enriched the GO terms of “RNA splicing and chromatin organization”, which may represent dermal cells that have received induction signals and are preparing for their subsequent differentiation. For gene set 3 we observed high expression of *Lef1*, *Twist2*, *Foxd1*, *Cntn4* (Figure 2C and Figure S3C), and these genes enriched the GO terms of “negative regulation of cell differentiation, organophosphate catabolic process, and regionalization”. It is of interest that *Lef1* and *Twist2* have been recently identified as pre-DC markers by Mok *et al*., and it is therefore plausible that gene set 3 represents a pre-DC stage signature gene list 7. After the pre-DC stage, our analysis revealed that DC fated cells showed a significantly high expression level of *Prdm1*, *Heyl*, *Tshz1*, *Sox18* (Figure 2C and Figure S3C), all of which were recently identified DC markers, further emphasized their DC identity. Furthermore, when evaluating cell cycle progression along pseudotime we also found that the DC cell branch mainly arrested at the G1 phase (Figure S3D), which was consistent with recent findings that cell cycle exit was a marker of acquisition of the DC fate 27. Immunofluorescence analysis also indicated that DC expressed PRDM1 and LEF1, but not PCNA, further emphasizing that DC cells exit the cell cycle at this stage (Figure 2D). The expression of CTNNB1 and BMP2 was also detected in the DC cell populations while KRT15 was mainly expressed in the epidermal cells. Taken together, our analysis here successfully recapitulated the* bona fide* dermal cell fate decision to a DC fate, and revealed the transcriptome landscape during DC fate commitment.

After recapitulation of the dermal cell transcriptome landscape during the hair follicle induction stage we then investigated the key TFs involved during the first dermal cell fate decision. We implemented a single-cell regulatory network inference and clustering (SCENIC) pipeline to infer key regulons involved during the hair follicle cell fate decisions (Figure S4) 37. The SCENIC algorithm revealed a series of key regulons and their corresponding target genes (Table S3). For the DC branch cells, they enriched regulons such as *Gli1*, *Hes1*, *Sox9*, *Foxd1*, *Foxp1*, *Sox18*, *Prdm1*, and *Sox2* (Figure 2E), all of which were also defined markers during DC fate commitment 7. Besides, comparison of SCENIC identified TFs with Mok *et al*., identified TFs showed ~30% overlap (Figure S3E). Our immunofluorescence analysis also verified the expression of SOX9 and SOX18 in the DC population (Figure 2D and Figure S3F). We also revealed other candidate regulons which may play vital roles during DC specification such as, *Glis2*, *Zfp160*, *Zmiz1,* and *Sox11,* etc. Combining our Monocle cell fate comparison assay with our regulon inferring assay we further analyzed sequential activation of the key regulons (Figure 2F) and it was revealed that *Sox2*, *Foxp1*, *Foxd1* were early activated regulons targeting to *Lef1*, *Foxd* family members, and *Foxo1*. Noteworthy, these regulons illustrated above and their corresponding targets have been recently identified as preDC signature genes (tick labeled).

### Pseudotime ordering analysis reveals that G/AA-DP and ZZ DP are transcriptional distinct branches

**Figure 3:**
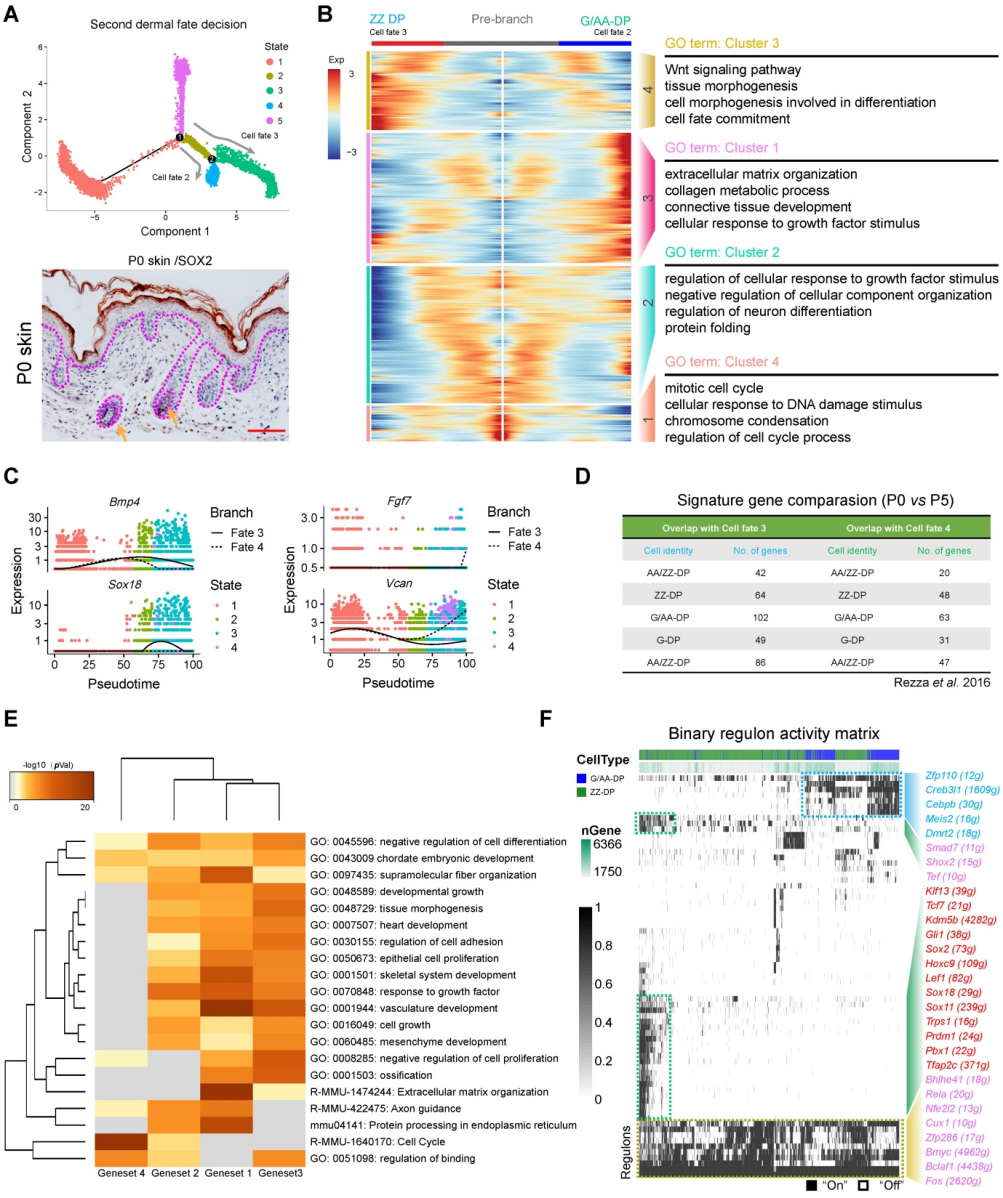
** Dissecting DP population heterogeneity in the late stage of hair follicle development.** (A) Monocle pseudotime trajectory construction analysis and immunohistochemistry analysis of SOX2 expression in P0 skin. Arrows indicate the SOX2 positive DP population. Scale bars, 25 µm. (B) Heatmap illustrating DP signature gene dynamics from pre branch to ZZ DP and G/AA-DP fate. The corresponding GO terms for each gene set were listed in the right panel. (C) ZZ DP makers *Bmp4*, *Sox18* expression and G/AA-DP markers *Fgf7*, *Vcan* expression along pseudotime. Cells were color-coded with cell states and the solid line represents cell fate 1, while the dashed line represents cell fate 2. (D) Comparison of DP signature genes in this study with previously identified different DP signature genes using bulk RNA seq. No. of genes depicts the number of overlapped genes. (E) Heatmap comparasion of enriched GO terms among 4 different gene sets. (F) SCENIC binary regulon activity heatmap deciphering G/AA-DP and ZZ DP branch specific enriched regulons. “On” depicts active, while “Off” represents inactive.

Since we successfully recapitulated the dermal cell lineage pseudotime differentiation trajectory and delineated the DC cell fate commitment prior to E16.5, we then focused on the next development trajectory from E16.5 to P0 (Figure 3A, top panel). This is the time corresponding to DP cells being specified, including guard hair follicles, Awl/Auchene hair follicles, and Zigzag hair follicles 23, 38. Interestingly, consistent with Driskell *et al*., our immunohistochemistry analysis showed that SOX2 was expressed in the G/AA-DP, but not the ZZ-DP (Figure 3A, lower panel). Pseudotime ordering analysis revealed that DP cells bifurcated into two branches, suggesting that two distinct DP populations exist in the P0 skin hair follicles (Figure 3A, top panel). To further identify signature genes between the different DP cell populations we used Monocle to perform differential gene expression analysis between the two branches (Figure 3B). Our analysis identified 606 signature genes for cell fate 3 and 1,004 signature genes for cell fate 4. Specifically, we found that branch 3 expressed high levels of *Bmp4*, *Sox18*, *Fgfr1*, *Gli1*, *Lef1*, and *Notch1*, while branch 4 showed high expression of *Fgf7*, *Vcan*, *Wnt9a*, *Notch1*, *Dlk1*, *S100b*, and *Sod3* (Figure 3C and Figure S5A). For cell fate 3, the differentially expressed genes (DEGs) enriched the GO terms of “Wnt signaling pathway, tissue morphogenesis, and cell morphogenesis involved in differentiation”, while for cell fate 4, DEGs enriched the GO terms of “extracellular matrix organization, collagen metabolic process, and connective tissue development”. Several groups demonstrated that *Sox18* specifically expressed in the zigzag hair follicle DP 39, 40, and the ablation of *Sox18* has been demonstrated to reduce zigzag hair formation 24, 25. We further performed mRNA *in situ* hybridization (ISH) to validate the expression of *Sox18* in different hair follicles and the result showed that *Sox18* mainly expressed in the zigzag hair follicles (Figure S5B). Therefore, we termed cell fate 3 as the ZZ-DP fate (Figure 3B and Figure S5B, left panel). For cell fate 4, Driskell *et al*. demonstrated that *Fgf7* significantly increased in G/AA-DP compared with the ZZ-DP 20, we therefore termed cell fate 2 as the G/AA-DP fate (Figure 3B and Figure S5C, right panel). Noteworthy, the well-known G/AA-DP *Sox2* was not detected in the current dataset; such inconsistency may be caused by the high number of zero counts of *Sox2* during sampling.

We then compared previously reported DP signature genes with our Monocle analysis identified G/AA-DP and ZZ-DP signature genes. As far as we know little transcriptome information is available for P0 DP from different hair follicles, we therefore compared our identified DEGs with previously identified DP signature genes from P5 hair follicles (Figure 3D and Table S4). Unexpectedly, it was found that DEGs from ZZ-DP or G/AA-DP overlapped similarly with previously identified 5 DP populations. However, it's notable that the branch endpoint showed similar expression of *Sox18*, *Bmp4*, and *Vcan*, while the differences mainly concentrated intermediate cells en route to the end branch (Figure S5C). It is therefore plausible that intermediate states may exist during the underappreciated DP specification stage *prior to* entering the hair cycle, which may also account for the inconsistent expression reported by different groups. We further visualized several core DP markers and also observed a similar expression pattern including, *Fgf7*, *Lef1*, *Gli1*, and *Notch1* (Figure S5D) 21.

To further capture the relationships between the terms enriched from the different gene sets we then compared enriched GO terms between different cell clusters (Figure 3E, Figure S5E). It was found that the four gene sets shared many co-enriched GO terms, and the top enriched GO terms included extracellular matrix organization, vasculature development and response to growth factors. We also applied SCENIC to infer key regulons involved during the DP cell fate decision (Figure 3F). It was found that both DP populations enriched regulons such as *Bmyc*, *Bclaf1*, and *Fos*, while the ZZ branch specifically enriched regulons such as *Gli1*, *Sox2*, *Lef1*, *Sox18,* and *Prdm1*, and the G/AA-DP branch enriched regulons such as *Zfp110*, *Creb3l1*, *Meis2*, and *Dmrt2*. Differences found between regulon enrichment also suggests the heterogeneity of the ZZ-DP and G/AA-DP, which may be responsible for the asynchronous development of different hair follicles.

### Recapitulating epithelial cell fate decisions towards matrix and IFE precursors (E13.5 - E16.5)

**Figure 4:**
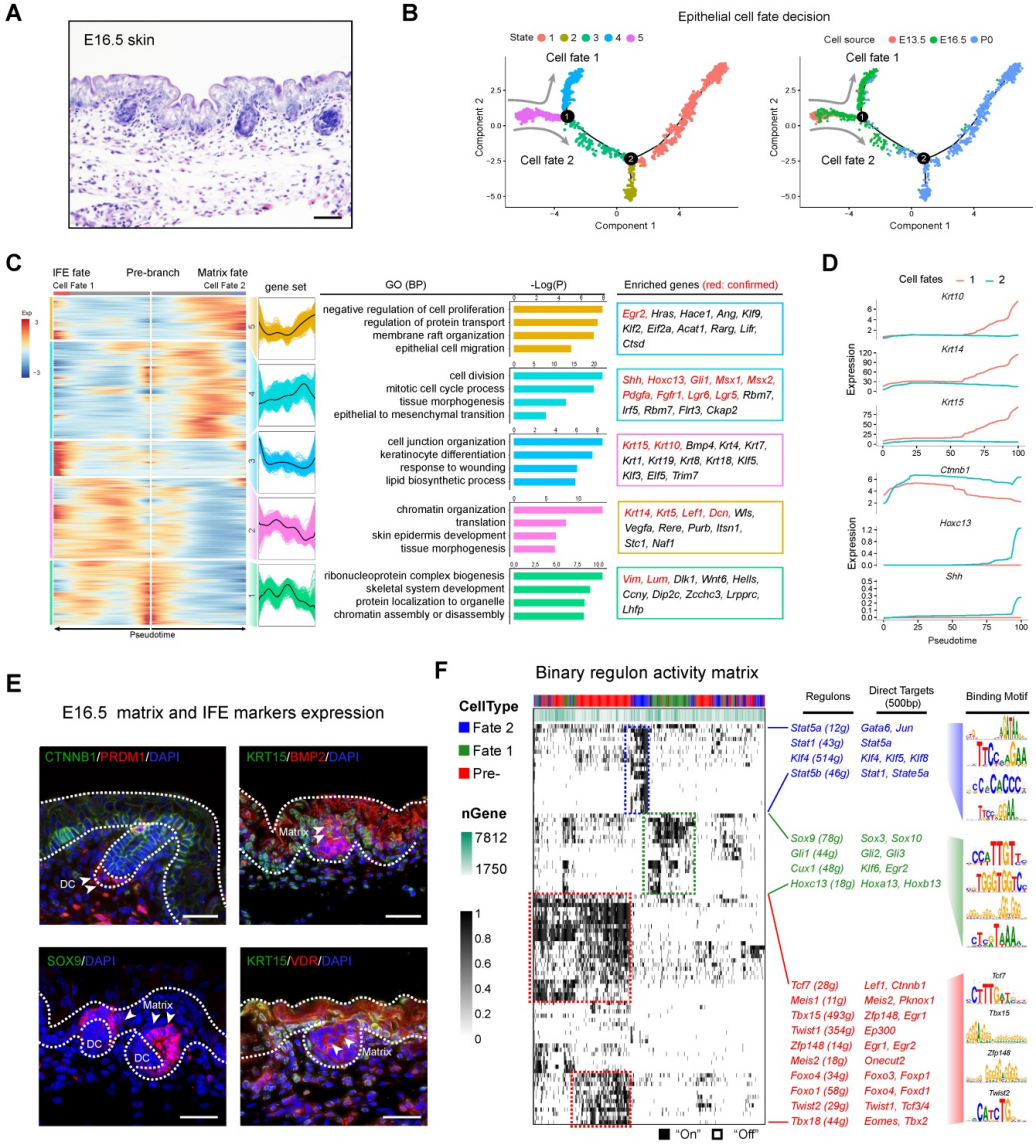
** Dissecting molecular signature underlying matrix and IFE precursor fate commitment.** (A) Histology analysis of E16.5 embryonic skin. Scale bar, 50 µm. (B) Pseudotime ordering of all epithelium cell populations from three developmental time stages. Each dot represents one cell. The left plot was color-coded with stage information, while the right plot was color-coded with developmental stages. (C) Heatmap displaying branch specific DEGs expression in branch point 1 in Figure 4B. Cell fate 1 indicates matrix fate and cell fate 2 indicates IFE fate. The corresponding expression curve and enriched GO terms for each gene set were visualized in the middle panel. The representative DEGs for each gene set were shown in the rectangular box (right panel) with red depicting confirmed signature genes. (D) Expression dynamics of representative branch specific marker genes along pseudotime. (E) Immunofluorescence analysis of CTNNB1, PRDM1, KRT15, BMP2, SOX9 and VDR expression in E16.5 skin tissues. Scale bar, 50 µm. (F) Binary regulon activity heatmap illustrating the branch-specific enrichment of key regulons. The representative regulons and their corresponding targets (500 bp upstream of TSS) were listed in the middle panel. The binding motif was listed in the right panel.

After delineating the dermal cell fate decisions we then investigated the underappreciated epidermal cell lineage fate decisions. Histology analysis showed that E16.5 mouse dorsal skin had obvious primary follicle and stratified epithelium structures (Figure 4A). We then extracted epithelial cells from Seurat and performed Monocle cell trajectory analysis using variable genes identified by Seurat as ordering genes (Figure 4B, left and Figure S6A-B). Cell trajectory analysis revealed that epithelium cells also showed two main bifurcation points along the cell trajectory. The first bifurcation point consists of cells derived from E16.5, while the second bifurcation point mainly consists of cells derived from newborn mouse dorsal skin (Figure 4B, right). RNA velocity analysis also showed obvious directional flow along the pseudotime trajectory (Figure S6C).

We initially focused on the first bifurcation point and performed DEG analysis comparing each branch (cell fate 1 *vs* cell fate 2 in Figure 4B). DEG analysis revealed the two branches as matrix progenitor cells and IFE cells as evidenced by the high expression of canonical markers (Figure 4C). For the IFE branch, the DEGs enriched two distinct gene sets (gene set 2 and 3) (Figure 4C). For gene set 2 they enriched genes such as *Krt14, Krt5, Lef1 and Dcn*, and enriched the GO terms of “chromatin organization, translation, and skin epidermis development”. For gene set 3, they enriched canonical IFE markers such as *Krt10*, *Krt15* and the GO terms “cell junction organization, keratinocyte differentiation, and response to wounding” 41. For the matrix cell populations our analysis also delineated 2 gene sets (gene sets 4 and 5) with the expression of classic matrix progenitor markers including, *Shh*, *Hoxc13*, *Gli1*, *Msx1*, *Msx2*, *Lgr5*, *Lgr6*, and *Pdgfra* 21, 42, 43. Similarly, immunohistochemistry analysis of SHH and HOXC13 also confirmed their expression in the P0 matrix cells (Figure S6D). GO enrichment demonstrated that matrix progenitors enriched the GO terms “cell division, tissue morphogenesis and epithelial to mesenchymal transition” for gene set 4 and “negative regulation of cell proliferation, membrane raft organization and epithelial cell migration” for gene set 5, respectively. We further compared the expression of conserved marker genes between the two cell fates and it was found that *Krt10*, *Krt14*, and *Krt15* similarly up-regulated along pseudotime in IFE cell fates but not the matrix cell fate, while *Ctnnb1*, *Hoxc13* and *Shh* showed higher expression in the matrix cell populations (Figure 4D), further confirmed their corresponding identities. Immunofluorescence analysis also confirmed the expression of CTNNB1 and KRT15 in the IFE cell populations, while BMP2, SOX9 and VDR showed higher expression in the matrix cell populations (Figure 4E), which was consistent with our analysis above.

Next, we performed SCENIC regulon inferring analysis and obtained a list of candidate cell state specific regulons involved in the matrix progenitor and IFE cell specification (Figure 4F). For the unspecified epidermis (state 5) our DEG analysis showed that *Lef1* showed high expression levels in the pre-branch gene set (Figure S6E), which consisted of all epithelial cells derived from the E13.5 dorsal skin. SCENIC analysis indicated that regulons such as *Tcf7*, *Tbx15*, *Foxo1*, *Foxo4* were enriched in the pre-branch gene set. Noteworthy, *Lef1* and *Ctnnb1* (*β-catenin*) were two direct targets of *Tcf7* and together with the fact that *Ctnnb1* knockout mice fail to form DC during hair follicle development 7, 44, 45, these data further emphasize the crucial role of the LEF/TCF/β-catenin signaling pathway in controlling DC formation. We also delineated a series of other regulons and their corresponding targets including *Tbx15* (targets to *Zfp148*, *Egr1*), *Zfp148* (targets to *Egr1*, *Egr2*), Foxo signaling members *Foxo4* and *Foxo1* and twist signaling members *Twist 1, 2* (target to *Tcf 3/4*, *Ep300*), all of which have been described in previous research. For the matrix progenitor branch our analysis demonstrated that *Sox9*, *Hoxc13*, *Gli1,* and *Cux1* were the core regulons involved, while for the IFE fate we delineated that *Stat* family members *Stat1*, *Stat5a*, *Stat5b* and Krüppel-like factor gene family members *Klf4*, *Klf5*, *Klf8* were the core identified regulons.

### Revealing hair shaft and IRS fate decisions during cytodifferentiation (E16.5 - P0)

**Figure 5:**
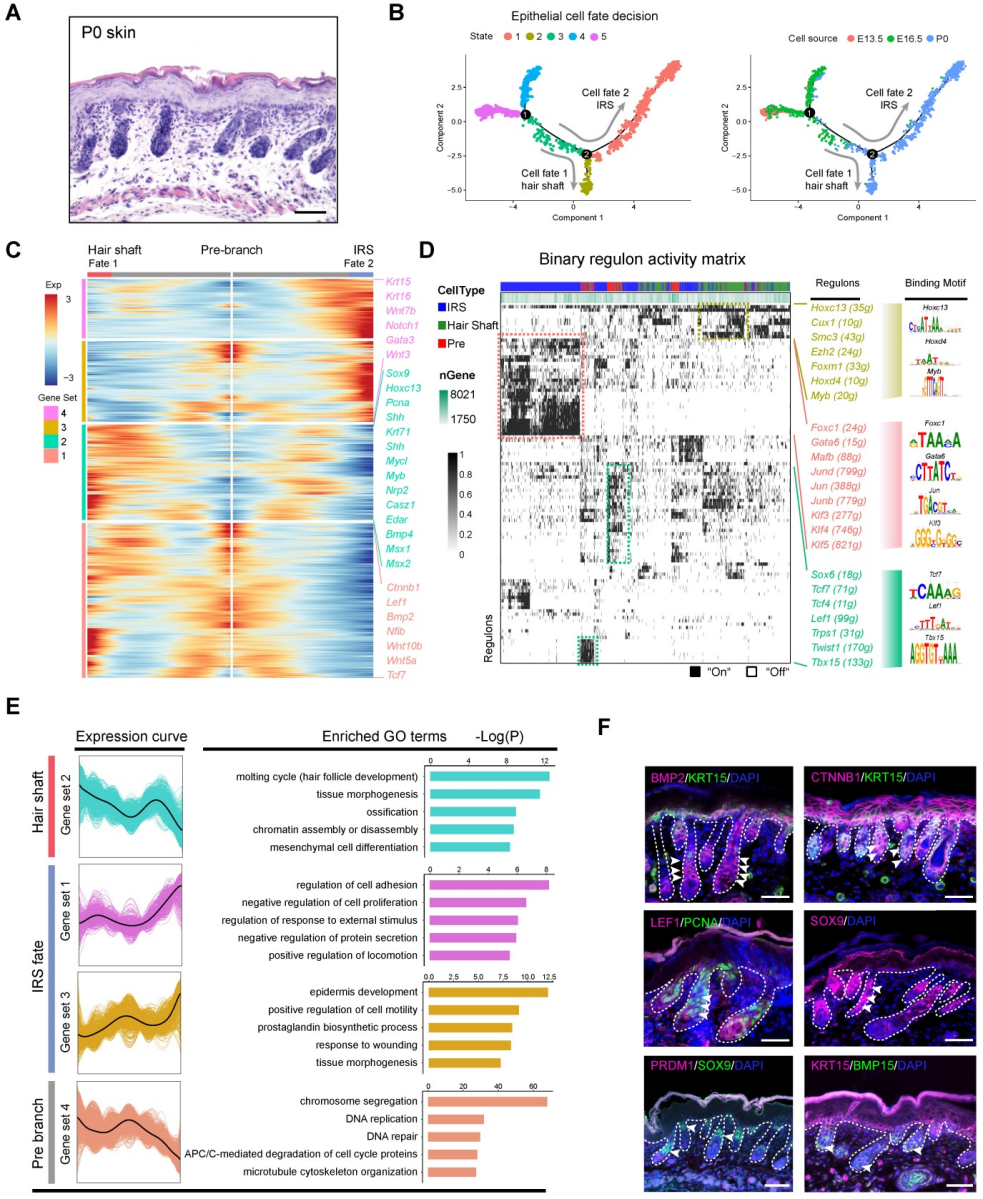
** Dissecting hair shaft and IRS fate commitment from matrix precursors.** (A) Histology analysis of P0 mouse skin. Scale bar, 50 µm. (B) Pseudotime visualization of the hair shaft and IRS fate decisions in the epithelium single cell pseudotime trajectory. (C) Heatmap illustrating branch specific DEGs dynamics along pseudotime. Cell fate 1 indicates hair shaft fate and cell fate 2 indicates IRS fate. (D) Binary regulon activity heatmap demonstrating branch specific enrichment of regulons. The cell states correspond to Figure 5 B. The representative regulon and binding motifs were listed in the right panel. (E) DEGs expression dynamics and GO enrichment in each gene set. The gene expression curve was listed in the left panel and GO terms for each gene set were listed in the right panel. (F) Immunofluorescence analysis of BMP2, CTNNB1, LEF1, SOX9, KRT15, PCNA, and BMP15 in P0 skin. Scale bar, 25 µm.

After recapitulating the key events involved at the hair follicle organogenesis stage (around E16.5), we then focused on the next cytodifferentiation stage (around P0) (Figure 5A). As expected single-cell trajectory analysis of the P0 epithelium cells bifurcated into two branches (Figure 5B). To infer their cell identity, we performed DEG analysis between the branches using Monocle. It was found that cell fate 2 significantly enriched IRS related marker genes including, *Gata3*, *Notch1*, *Krt16*, *Wnt7b* and *Scube2*, while cell fate 1 enriched hair shaft related genes, including *Lhx2*, *Shh*, *Hoxc13*, *Mycl*, *Myb*, *Nrp2*, *Casz1*, and *Edar* (Figure S7A) 46. These data together demonstrated that we have successfully recapitulated the hair shaft and IRS differentiation trajectory from the matrix progenitors. To further unmask the gene expression profile and gene regulatory network underlying hair shaft and IRS specification we performed DEG analysis, and SCENIC regulon inferring analysis, along with the Monocle trajectory analysis (Figure 5C, D). Monocle branch specific gene expression analysis revealed hair shaft enriched genes such as *Shh*, *Hoxc13*, *Msx1/2,* and *Bmp4*, all of which had been well characterized to play vital roles during hair shaft differentiation 8, 46. We also found a series of other DEGs such as *Krt25*, *Krt71*, *Mycl*, *Myb* and *Lhx2*. We further compared our Monocle identified DEGs with Anagen VI hair shaft/IRS signature genes identified by Yang *et al*. (Figure S7B). We identified 585 branch specific DEGs for hair shaft and about 3.76 % (22/585 genes) overlapped with the anagen VI hair shaft signature genes, thus it is plausible that the hair shaft cells display distinct gene expression patterns prior to entering the hair cycle (Table S5). Similarly, the IRS gene set 4 shared 2.75 % (10/363) and IRS gene set 3 shared 3.23 % (16/495) overlapping genes with the anagen IRS signature genes. These data together emphasized that the embryonic hair shaft and the IRS shared distinct gene expression profiles compared with the anagen hair shaft and the IRS.

We then used SCENIC to infer transcription factor (TF) regulatory information among the three branches (Figure 5D). SCENIC regulon inferring analysis revealed that the hair shaft branch (State 2) enriched regulons such as *Hoxc13*, *Cux1*, *Hoxd4*, and *Myb*. The expression of *Hoxc13* had been long demonstrated as a key transcription factor in promoting hair shaft specification. Our analysis here also unmasked a series of significantly enriched regulons for the underappreciated IRS (State 1) which included *Gata6*, *Foxc1*, *Jun* family members (*Jun*, *Junb*, *Jund*), and *Klf* family members (*Klf3/4/5*). Noteworthy, *Gata6* has been previously demonstrated as a marker for the IRS and perturbation of *Gata6* caused dilation of the hair follicle canal demonstrating an indispensable role during hair follicle morphogenesis 47.

To gain further insight into the gene regulatory machinery underlying the matrix progenitors' commitment to the hair shaft or IRS we compared their gene expression profiles along the pseudotime trajectory (Figure 5E). We first compartmentalized the identified branch specific DEGs using k-means clustering. The hair shaft significantly enriched gene clusters (cluster 2) enriched GO terms of “molting cycle (hair follicle development), tissue morphogenesis, and ossification”. Previously, Yang *et al*. demonstrated that signature genes of anagen VI hair shaft had the enriched GO terms of “cholesterol biosynthetic process, steroid biosynthetic process and lipid metabolic process, which further emphasized that the hair shaft showed distinct gene expression patterns prior to entering the hair cycle. Similarly, the IRS highly expressed genes (gene set 3, 4) had the enriched GO terms “regulation of cell adhesion, negative regulation of cell proliferation (gene set 1) and epidermis development, positive regulation of cell motility, and prostaglandin biosynthetic process (gene set 3),” which were also different from Yang *et al*. which had the anagen IRS signature gene enriched GO terms “DNA replication, cell cycle, and multicellular organism development. Last, consistent with our analysis, our immunohistochemical staining assay confirmed the expression of HOXC13 and SHH in the hair shaft (Figure S6D), while KRT15 highly expressed in the IRS (Figure 5F). Furthermore, we found that the hair shaft showed a high expression of the cell proliferation marker PCNA while BMP15 was mainly expressed in the DP cells, and PRDM1 was not detectable in hair follicles at P0. Taken together, our analysis here provides underappreciated information during the matrix cell fate commitment to the hair shaft and IRS.

### Ligand-receptor interaction prediction during hair follicle morphogenesis

**Figure 6:**
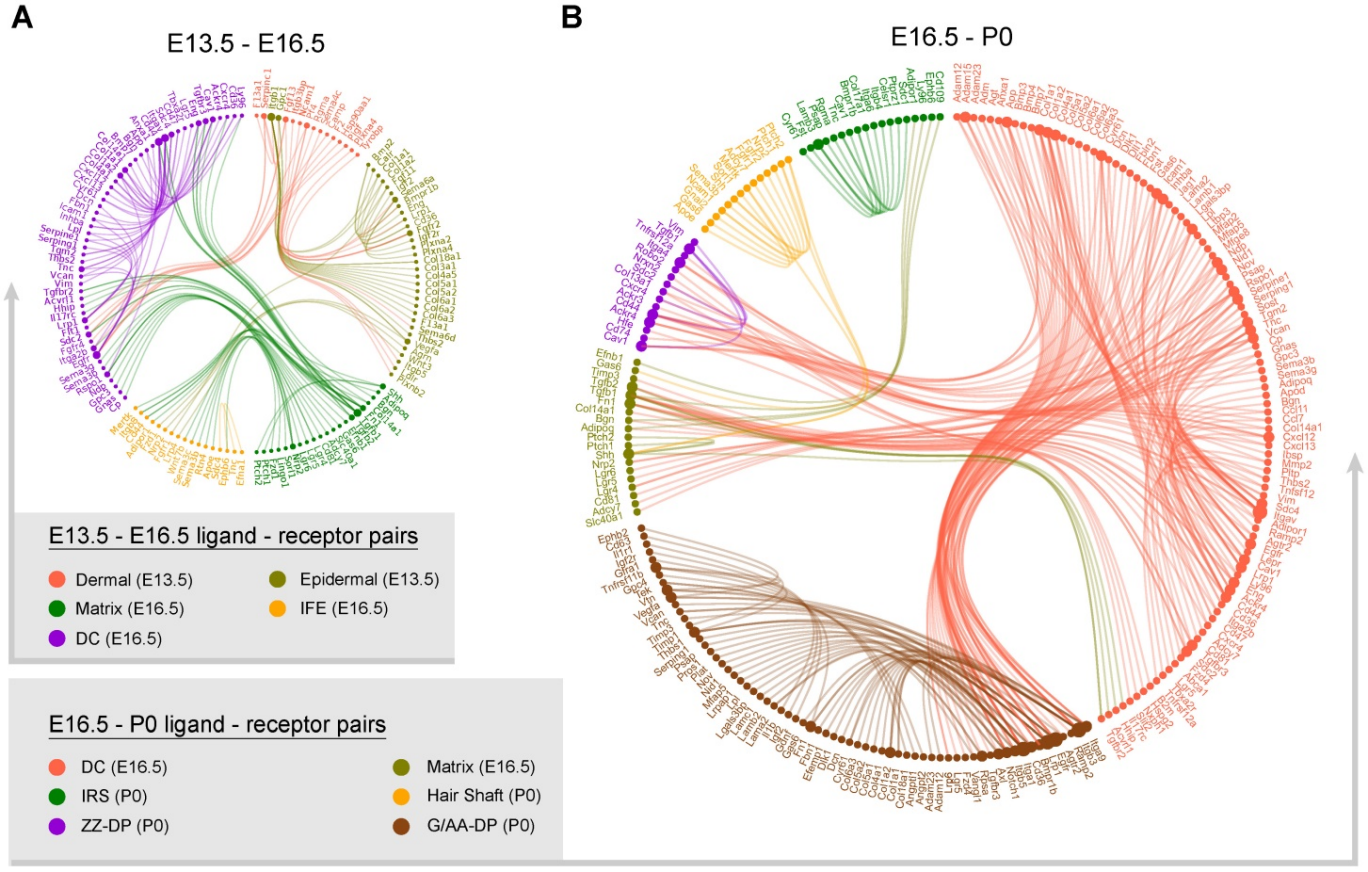
**Intercellular ligand-receptor prediction.** Ligand-receptor pairs between E13.5-E16.5 (A) and E16.5-P0 (B) main cell populations. Different cell populations were color-coded and ligand-receptor pairs were linked with a solid line.

Since we have successfully recapitulated dermal and epidermal cell fate decisions and delineated the molecular signatures of the different cell populations we then used a public ligand-receptor database to infer intercellular communications during early hair follicle development 48, 49. By comparing the cell identity specific genes with ligand-receptors, we sorted hypothetical ligand-receptor pairs among different cell populations (Figure 6). For E13.5 to E16.5 ligand-receptor pairs (DC specification and epidermal specification to the matrix and IFE) we found stronger interaction relationships amongst the matrix, IFE, and DC populations at E16.5 (Figure 6A). We observed robust ligand-receptor pairs within the DC population including, *Vcan*, *Egfr*, and *Bmp7* indicating a strong autocrine relationship at this stage. In the E13.5 dermal and epidermal cell populations we also observed strong intercellular communication.

At the hair follicle cytodifferentiation stage, we inferred potential ligand-receptor pairs using priori knowledge. Our analysis showed strong intercellular communication between the E16.5 DCs and the P0 G-DP cells including the BMP signaling members *BMP3*, *4*, and *7*, Notch signaling ligands *Jag1*, *Dlk1,* and collagens family members *Col1a1*, *Col1a2*, *Col4a1*. This further emphasized the indispensable roles of these well-defined pathways during DP specification. We also observed strong intercellular communication between the E16.5 DC and the P0 ZZ-DP cells. However, some of these ligand-receptor pairs differ between the E16.5 DC and the P0 G-DP, further suggesting heterogeneity in DP cells as we illustrated in our DEG analysis. For IRS, hair shaft and G-DP, we also observed strong autocrine signaling as revealed by abundant ligand-receptor pairs within the same cell population. These analyses together showed that strong intercellular communication was involved during early hair follicle development.

## Discussion

The lack of bona fide markers characterizing key cell populations, and the asynchronous development of different hair follicles, during early development has produced major obstacles hindering our current understanding of hair follicle development. However, seminal works have revealed the molecular signatures of different hair follicle cells and their roles during hair follicle development. Although the conclusion raised by different groups varies, two major questions remain to be answered: First, the *bona fide* characterization of molecular signatures of key cell populations involved during hair follicle morphogenesis and second, the level of heterogeneity within DP populations, which may be responsible for the asynchronous growth of the hair follicles.

With the development of high-throughput scRNA-seq analysis many complicated biological processes have been delineated in an unprecedented manner. This has been particularly beneficial in the area of organogenesis research as scRNA-seq has the robust ability to deconstruct cell heterogeneity within complicated tissues 50. Very recently, by using scRNA-seq two back-to-back papers published in *Developmental Cell* have delineated an underappreciated intermediate pre-DC fate transition stage occurring prior to DC formation (induction stage, E13.5 - E14.5) 7, 27. Here, utilizing the same technology we performed scRNA-seq on hair follicles at three-time points that encompass all three stages during hair follicle development (induction, organogenesis, and cytodifferentiation). By utilizing pseudotime trajectory construction, we for the first time revealed the dermal and epidermal cell lineage differentiation trajectories at single-cell resolution. Noteworthy, regulon activity scoring assay across dermal and epidermal cell populations revealed cell-type conserved gene regulatory network, which was consistent with tSNE clustering analysis. Besides, the regulon activity scoring assay also revealed that different cell states along the pseodotime trajectory also showed different enrichment of regulon subnetworks, thus deciphering their roles on cell fate commitment. Although most of the cell-type-specific gene regulatory networks remain largely to be verified, our analysis here provides an important resource and guide for the future functional validation. Furthermore, considering the fact that intercellular communication plays vital roles in cell fate commitment during hair follicle development, we also performed ligand-receptor interaction prediction during hair follicle morphogenesis and observed many ligand-receptor pairs among the different cell populations at different developmental timepoints. Although this information remains largely elusive we believe that this information is biologically important and may provide insights for understanding the intercellular interactions during hair follicle development.

For DC fate commitment in our analysis, we observed two different gene sets en route to a DC fate (Figure 2C, gene set 1 and 4). Interestingly, *Lef1* and *Twist2* were enriched in gene set 4, while *Prdm1* and *Sox18* were enriched in gene set 1. This was consistent with Mok* et al*., with the transcription factors *Lef1* and *Twist* expressed at the pre-DC stage 7 while the transcription factors *Prdm1* and *Sox18* were expressed at the DC1 stage, thus deciphering the bona fide characterization of an intermediate DC stage. We also identified other signature genes including *Tshz1* (a transcriptional regulation of developmental processes), *Heyl* (an effector of Notch signaling and a regulator of cell fate decisions), and *Cntn4* (a glycosylphosphatidylinositol-anchored neuronal membrane protein) which may function as new markers for these DC cell populations. Our analysis here is also consistent with recent findings that DC fate commitment requires cell cycle exit as evidenced by the upregulated expression of the cell cycle inhibitor *Btg1* and the downregulated expression of the pro-proliferative gene *Pcna* 16. Besides, when evaluating cell cycle progression along pseudotime, our data here showed that substantial dermal cell populations at E13.5 arrested at the G1 phase, further confirmed the hypothesis proposed by Gupta et al., that DC cells became quiescent as early as E13.5. These together further emphasize the presence of intermediate DC stages and that cell cycle exit is a marker event during DC specification.

We also delineated the epithelium fate commitment to IFE and matrix precursors which to our knowledge has not been comprehensively reported 8. By using Monocle pseudotime ordering analysis we successfully recapitulated the IFE and matrix cell differentiation trajectory and found that matrix cells at this stage-enriched genes such as *Shh*, *Pdgfra*, *Gli1*, *Hoxc13*. Interestingly, the expression of *Shh*, and *Pdgfra* in matrix cells has been demonstrated to promote down growth via *Gli1*, *Gli2* 11 which was consistent with our analysis here. Our GO analysis revealed that “hair follicle development, tissues morphogenesis” were enriched in matrix signature genes, further deciphering the molecular events during the envelopment of the DC cells by the matrix cell populations. Interestingly, the expression of *Hoxc13*, a key component involved in hair shaft differentiation, had been detected as early as E16.5 indicating an earlier activation of hair shaft fate than previously understood. It would be interesting to investigate whether these intermediate states exist during hair shaft fate commitment in future studies. Our analysis showed that IFE fate commitment involved canonical IFE markers such as *Krt10, Krt14* and *Krt15*. DEG analysis revealed 2 gene sets (gene set 4 and 5 in Figure 4C) during matrix cell fate commitment, which were reminiscent of the intermediate preDC stage prior to DC fate commitment. For gene set 4, DEGs enriched the GO terms “cell division, mitotic cell cycle process and tissue morphogenesis”, while gene set 5 enriched the GO terms “negative regulation of cell proliferation, regulation of protein transport and membrane raft organization”, displaying distinct molecular pathways are involved. Therefore it is plausible that the intermediate cellular stages may exist during IFE fate commitment and future studies may focus on this topic.

As development continues matrix cells give rise to the hair shaft and IRS populations, which was consistent with our Monocle analysis here (Figure 5B). There is limited information regarding the molecular signatures of key cell populations in the late two stages of hair follicle development. Here we successfully delineated hair shaft and IRS signature gene expression profiles and the gene functional categories involved during their fate commitment from matrix precursors. For hair shaft fate commitment our analysis showed that *Hoxc13*, *Shh*, *Bmp4*, *Msx1/2* were specifically enriched, which was consistent with previous findings 8. However, the expression of *Notch1* and *Lef1* was not enriched in cells with a hair shaft fate but in cells with the IRS fate and the pre branch fate, respectively. For GO enrichment, hair shaft had enriched GO terms involved in molting cycle, hair cycle, and hair follicle development. This showed distinct differences compared with the anagen hair shaft, thus emphasizing distinct gene expression profiles after entering the hair cycle. Interestingly, for IRS fate specification, our analysis also observed two gene sets. Gene set 1 enriched the GO terms “regulation of cell adhesion and regulation of cell-cell adhesion” and gene set 3 enriched the GO terms “epidermis development and skin development”. This may also indicate different intermediate stages prior to IRS fate commitment. Taken together, our data here provides an unprecedented insight into the gene expression profiles of hair shaft and IRS cell populations during the late stages of hair follicle development. More importantly, the heterogeneity within these particular cell populations was revealed in our research and may also account for the discrepancy in gene expression signatures previously reported.

Unexpectedly, pseudotime ordering of all dermal cell lineages revealed two branch points particularly for the later stages (E16.5 - P0) which was reminiscent of the discussion of DP heterogeneity. In 2009, Driskell *et al*. showed that different signaling is involved during hair follicle type determination 20, while subsequent research by Chi *et al*. demonstrated that the number of DPs dictated the size and shape of the hair 26. Further enhancing the latter hypothesis, Rezza *et al*., isolated different DP populations from P5 mice back skin based on a flow cytometry analysis and performed bulk RNA seq on different DP populations 21. By comparing their expression profiles, they demonstrated high similarities for all DP populations. However, our analysis here indicated that DP cell populations bifurcated into two distinct fates indicating DP heterogeneity. Noteworthy, for the discrepancy between reports by Driskell and Chi it should be mentioned that Chi *et al*. used adult mice as a research model in which hair follicles had entered the hair cycle, which may be different from an *in uter*o situation. Supporting such a hypothesis, our data here indicated that the IRS and hair shaft showed distinct gene expression patterns compared to those that have entered the hair cycle. Interestingly, by in-depth analyzing the DEGs between the two DP branches we found that the endpoint in each branch shared a higher similarity while the intermediate cells en route displayed high heterogeneity. It's therefore plausible that underappreciated intermediate stages may exist during DP development. Because of this, DPs showed similar molecular profiles in P5 as reported by Rezza *et al*. while displaying higher heterogeneity in DPs as reported by Driskell *et al*., in the embryonic skin. It will be interesting for future studies to focus on this topic which may provide new insights into hair follicle development.

In summary, our research highlights the characterization of the underappreciated molecular signatures of embryonic hair follicle progenitors using unbiased scRNA-seq. Based on single-cell transcriptome analysis we delineated key events underlying dermal and epithelium fate decisions during hair follicle morphogenesis. Our data here also provides new insights into the heterogeneity of the DP cell populations and intercellular communication during hair follicle development. These data together enable an in-depth understanding of the molecular machinery underlying embryonic hair follicle development.

## Methods

### Experimental Animals

About 7-8 weeks old C57/BL6 strain mice were used in this study, all mice used were purchased from Beijing Vital River Laboratory Animal Technology Co., Ltd and were housed in a temperature-controlled room with *ad libitum* food and water. To obtain pregnant mice, female mice were mated with male mice overnight at a ratio of 3:1, and mice with the presence of vaginal plug the next morning were denoted as 0.5 day post coitum (dpc). To obtain E13.5 and E16.5 fetal dorsal skin, the pregnant mice were sacrificed by cervical dislocation and the gender was determined according to the fetus ovary/testis. Male mice were used to avoid potential hair cycle variation as previously indicated 46. For each group, pooled skin samples isolated from at least three mice were used for sequencing library construction. All experimental procedures involved in this study were approved by the Experimental Animal Manage Committee of Northwest A & F University.

### Histological analysis and Immunofluorescence Staining

The skin tissues isolated from the fetal dorsal skin were fixed with 4 % paraformaldehyde (Sorlabio, Beijing, China) at 4 °C overnight. The next morning, the fixed tissues were then dehydrated in an ethanol solution and further incubated with xylene for 30 min. After incubation with xylene the samples were embedded in paraffin blocks. The embedded paraffin blocks were cut with a Leica RM2255 microtome (Leica, Nussloch, Germany) at a thickness of 5-7 μm and the samples were transferred to APES (ZSGB-BIO, Bejing, China) treated slides to avoid detachment.

For hematoxylin and eosin (H & E) staining, the slides were deparaffinized in 100% xylene solutions for 30 min and further rehydrated in an ethanol series. After rehydration the slides were stained with hematoxylin solution for 7 min followed by washing twice with distilled water for 5 min. After rinsed with 1 % HCl (v/v) ethanol solution for 3-5 s, the slides were immediately washed with 45 °C water for 5 min. Followed by a dehydrated procedure, the slides were then stained with 1% eosin ethanol solution and further rinsed with absolute ethanol solution for 10 min. Finally, the slides were mounted with neutral resins mounting medium and pictures were taken under an optical microscope.

For immunofluorescence staining analysis, the slides were deparaffinized in 100 % xylene solutions for 30 min and then hydrated with an ethanol series. To perform antigen retrieval, slides were incubated in boiled 0.01 M sodium citrate buffer (pH = 6.0) for 10 min and then cooled down to room temperature. Blocking was performed with 3 % BSA and 10 % donkey serum in 0.5 M Tris-HCI buffer for 30 min at room temperature, and slides were then incubated with primary antibodies at 4 °C overnight.

The primary antibodies and secondary antibodies used in this study were listed in Table A.

The next morning, the slides were further incubated with secondary antibodies at 37 °C for 30 min. DAPI was used to stain nuclei and the slides were mounted with anti-fade mounting medium. Pictures were taken under LEICA TCS SP5 II confocal microscopy (Leica Microsystems GmbH, Wetzlar, Germany). For enzyme substrate-based immunohistochemistry, the slides were washed with 3 % H_2_O_2_ for 10 min to block endogenous peroxidase activity prior to blocking and DAB (ZSGB-BIO, Bejing, China) solution was used for the chromogenic reaction.

### *In situ* hybridization

To detect the *Sox18* mRNA molecule in P0 skin tissues, we used a commercially available kit from GenePharma (GenePharma, Shanghai, China). Briefly, the sections were firstly deparaffinized in 100% xylene solutions for 20 min, protease K was then incubated with the slides at 37 °C for 20 min. After denaturation at 78 °C for 8 min, the CY3-conjuncted probe was then hybridized with the samples at 73 °C for 5 min and the sections were incubated at 37 °C overnight. The next morning, DAPI was added to stain the nuclei and the slides were mounted with anti-fade mounting medium. Pictures were taken under a fluorescent microscope.

### Single-cell suspension preparation

For each group skin tissues were obtained from at least 6 independent male fetuses prior to digestion. To prepare dorsal skin single cell suspension for single-cell RNA sequencing, the fetus back skin tissues were isolated via microdissection and 0.25 % trypsin/EDTA solution was used to digest E13.5 and E16.5 fetus dorsal skin tissues at 37 °C for 5 min. For P0 mice dorsal skin tissues, 2 mg/ml collagenase IV (Sigma, St Louis, MO, USA) was used to digest skin tissues at 37 °C for 30 min. After trypsinization, the skin tissues were mechanically dissociated into single cell suspension by pipetting, the cell suspensions were then filtered through a 40 μm nylon cell strainer (BD Falcon, BD Biosciences, San Jose, CA, USA) prior to single cell library construction.

### Single-cell library preparation and sequencing

Single cell barcoding and library preparation were performed based on 10×Genomics single-cell RNA sequencing platform (10×Genomics, Pleasanton, CA, USA). Briefly, the single cell suspension prepared above were immediately counted using a hemocytometer (TC20, Bio-Rad, Hercules, CA, USA) and the cell concentrations were adjusted to 1,000 cells/μl prior to barcoding. To barcode the single cells with 10×Barcoded gel beads, 10×Genomics Chromium Single Cell 3' Library & Gel Bead Kit v2 (10×Genomics Inc., Pleasanton, CA, USA, 120237) and 10×Genomics Chromium barcoding system was used to construct 10×barcoded cDNA library following the manufacturer's instructions. Illumina HiSeq X Ten sequencer (Illumina, San Diego, CA, USA) was used for sequencing and pair-ended 150 bp (PE150) reads were generated for downstream analysis.

### 10x sequencing data preprocessing

The CellRanger (v2.2.0) software was used for analyzing raw sequencing data according to 10x Genomics official pipeline (https://support.10xgenomics.com/single-cell-gene-expression/software/pipelines/latest/what-is-cell-ranger). Briefly, we firstly used CellRanger 'cell ranger count' wrapped function with '--expect-cells=5000' argument to evaluate the total number of cells captured in each data set (24,805 cells in E13.5 group, 7,250 cells in E16.5 group and 9,021 cells in P0 group, respectively), then, the generated FASTQ files were processed with '--force-cells = 7000' argument to make them easier to compare after integration. Cell ranger count function used wrapped STAR software to align sequence to the reference genome and the 10x pre-built mouse genome (mm10-3.0.0) was used (https://support.10xgenomics.com/single-cell-gene-expression/software/downloads/latest). The output files containing gene expression matrices and barcode information of CellRanger pipeline were then used for downstream visualization analysis.

### Characterization of cell clusters

After CellRanger pipeline, the quality control (QC) and cell clustering were analyzed with single-cell RNA seq Seurat software (v2.3.4) based on R environment (R version: 3.5.1, https://www.r-project.org/) following the online guide (https://satijalab.org/seurat/). We used 'filtered_gene_bc_matrices' files generated by CellRanger as input files for Seurat. For each dataset, we firstly filtered cells with unique detected genes less than 200 and genes detected less than 3 cells, then we used 'FilterCells' function to remove cells with a total number of detected genes (nGenes) less than 1,750. After normalization, the variable genes for each dataset were calculated for downstream clustering assay.

To compare transcriptome profiles along three different developmental timepoints, we then merged three different datasets using 'RunMultiCCA' function implemented in Seurat. RunMultiCCA used a canonical correlation analysis to remove variation caused by sample source. After dataset alignment, we then performed a clustering analysis on the integrated dataset based on tSNE algorithm implemented in Seurat. To identify cluster specifically expressed genes, we used Seurat implemented 'FindAllMarkers' function to calculate cluster markers and the tSNE identified cell clusters were annotated with based on the previously reported canonical marker genes expression.

To subcluster cell clusters of interest for in-depth analysis and/or downstream differentiation trajectory construction, we used Seurat implemented 'SubsetData' function to extract cluster of interest. The extracted subclusters were then re-run the Seurat pipeline, which provides higher resolution for dissecting cellular heterogeneity among particular cell types.

### Constructing single-cell pseudotime differentiation trajectory

To interpret cell differentiation fate decisions, we used Monocle (v 2.10.0) to order single cells along pseudotime according to the official tutorial (http://cole-trapnell-lab.github.io/monocle-release/docs/#constructing-single-cell-trajectories). To perform pseudotime ordering to particular cell types, we firstly subclustered interested cell type from Seurat object, then, the Monocle object was constructed using 'newCellDataSet' function in Monocle. To order single cells along pseudotime, we used Seurat identified variable genes as ordering genes to construct single cell differentiation trajectory. The root state was set according to cell Seurat identified cell cluster label and 'BEAM' function was used to calculate branch-specific expressed genes. To plot branch-specific expression heatmap, we used Monocle implemented 'plot_genes_branched_heatmap' function and genes with qval < 1e-4 were regarded as input genes. Gene clusters were further divided into four clusters according to k-means. To investigate gene functions in each gene clusters, we used Metascape (http://metascape.org/gp/index.html#/main/step1) to perform gene ontology (GO) analysis.

### RNA velocity analysis

RNA velocity analysis was performed according to the developer's instructions (http://velocyto.org/) 36. Briefly, velocyto.py script (http://velocyto.org/velocyto.py/tutorial/index.html#getting-started) was firstly used to generate unspliced and spliced mRNAs directely from 10×Cellranger output files, and the generated loom files were then processed with standard velocyto.R pipeline. To estimate RNA velocity, we firstly filtered cells that were not used in the pseodotime ordering analysis, and extracted embedding from Monocle object for downstream cell-cell distance calculation. Finally, the cauculated velocity vector fields were visualized on the Monocle embedding using 'show.velocity.on.embedding.cor' function.

### Core TFs prediction

To infer core TFs within each branch identified by Monocle, we used SCENIC algorithm to infer regulon activity within each cell states. The analysis pipeline was performed following the developer's instructions (https://github.com/aertslab/SCENIC). Briefly, the expression matrix was firstly extracted from Seurat and was further transformed into SCENIC required format (rows represent genes, columns represent cells) in R, we then extracted cellular branch information identified by Monocle to construct expression matrix of desired states. We further filtered cells with genes that were detected in less than 1% of the total cells and genes with less than at least 6 UMI counts across all samples. Then, GENIE3 was used to identify co-expressed gene modules and infer potential TF targets for each module based on the expression matrix. After that, RcisTarget was used to perform cis-regulatory motif analysis, we scanned two motif to TFs databases (mm10__refseq-r80__10kb_up_and_down_tss and mm10__refseq-r80__500bp_up_and_100bp_down_tss; https://resources.aertslab.org/cistarget/) and kept modules with significant motif enrichment, this modules were then termed as regulons according to SCENIC pipeline. To visualize regulon activity within each cellular state identified by Monocle, we further used SCENIC pipeline to binarize the regulon network activity based on AUCell algorithm, and we used binary regulon activity matrix to visualize regulon activity within each cellular state.

### Cell to cell ligand-receptor interaction analysis

To infer the hypothetical intercellular communication, we compared cell type-specific DEGs identified by Monocle and manually sorted ligand-receptor pairs according to cell relationship. The mouse ligand-receptor pairs used here were compiled by Skelly *et al.* and about 2,009 ligand-receptor pairs were used in this study 49. To reveal potential ligand-recepto pairs, we linked cells types when defined ligand-receptor pairs were expressed in both cell types, respectively. After sorting ligand-receptor pairs, we used igraph (https://github.com/igraph/rigraph) and edgebundleR (https://github.com/garthtarr/edgebundleR) R packages to visualize the ligand-receptor networks and the node represents genes while the solid line connects each ligand-receptor pair. To avoid confusion, we separately plotted E13.5 to E16.5 and E16.5 to P0 ligand-receptor pairs. Only cell types at the same time point and shares differentiation relationship were considered to sort ligand-receptor pairs.

## Figures and Tables

**Table A:** primary antibodies and secondary antibodies

Rabbit monoclonal anti-LEF1	Cell Signaling	Cat# 2286, RRID:AB_659971
Rabbit polyclonal anti-CTNNB1	Proteintech	Cat# 51067-2-AP, RRID:AB_2086128
Rabbit anti-VDR	Proteintech	Cat# 14526-1-AP, RRID:NA
Rabbit polyclonal anti-SOX2	Proteintech	Cat# 11064-1-AP, RRID:AB_2195801
Rabbit polyclonal anti-BMP4	Proteintech	Cat# 12492-1-AP, RRID:AB_2063531
Rabbit polyclonal anti-SHH	Proteintech	Cat# 20697-1-AP, RRID:AB_10694828
Mouse monoclonal anti-K15	Abcam	Cat# ab80522, RRID:AB_1603675
Rabbit monoclonal anti-BMP2	Abcam	Cat# ab214821; RRID:N/A
Mouse monoclonal anti-PCNA	Santa Cruz	Cat# sc-25280, RRID:AB_628109
Mouse monoclonal anti-SOX18	Santa Cruz	Cat# sc-166025, RRID:AB_2195662
Rabbit polyclonal anti-HOXC13	Sigma	Cat# HPA051634, RRID:AB_2616459
Rat monoclonal anti-PRDM1	Thermo Fisher	Cat# 14-5963-80, RRID:AB_1907438
Rabbit polyclonal anti-SOX9	Millipore	Cat# AB5535, RRID:AB_2239761
Donkey Anti-Rabbit IgG H&L (Alexa Fluor® 555)	Abcam	Cat# ab24273; RRID:AB_778767
Goat Anti-Mouse IgG H&L (Alexa Fluor® 488)	Abcam	Cat# ab150113; RRID:AB_2576208
HRP-labeled Goat Anti-Rat IgG(H+L)	Beyotime	Cat# A0192; RRID:NA
HRP-labeled Goat Anti-Rabbit IgG(H+L)	Beyotime	Cat# A0208; RRID:NA
HRP-labeled Goat Anti-Mouse IgG(H+L)	Beyotime	Cat# A0216; RRID:NA
