# Supplementary material for: Single-cell Transcriptome Profiling reveals Dermal and Epithelial cell fate decisions during Embryonic Hair Follicle Development
Source: Theranostics. 2020 Jun 12;10(17):7581–98. doi: 10.7150/thno.44306 (PMC7359078; doi:10.7150/thno.44306)
Supplement: Supplementary file 1 — Supplementary figures and tables. [file thnov10p7581s1.zip › ###2020.5.28_Ge's_Supplementary_Figures_compressed.pdf]

Single-cell transcriptome profiling reveals dermal and epithelial cell fate decisions during embryonic hair follicle development

Wei Ge [1], Shao-Jing Tan [2], Shan-He Wang [1], Lan Li [2], Xiao-Feng Sun [2], Wei Shen [2,¶], Xin Wang [1,¶]

1. Key Laboratory of Animal Genetics, Breeding and Reproduction of Shaanxi Province, College of Animal Science and Technology, Northwest A&F University, Yangling, Shaanxi 712100, China;

2. College of Life Sciences, Qingdao Agricultural University, Qingdao 266109, China.

¶ Correspondence and reprint requests to:

Prof. Wei Shen; E-mail: wshen@qau.edu.cn; shenwei427@163.com

Prof. Xin Wang; E-mail: wxwza@126.com

Supplementary Figure 1

A

Sample info.	E13.5	E16.5	P0
Estimated Number of Cells	7,000	7,000	7,000
Valid Barcodes	97.2%	97.3%	97.6%
Mean Reads per Cell	75,538	77,370	75,560
Median Genes per Cell	2,434	2,978	1,989
Total Genes Detected	19,997	19,767	19,145
Reads Mapped to Genome	90.5%	91.5%	94.0%
Reads Mapped Confidently to Transcriptome	62.8%	65.2%	71.7%

B

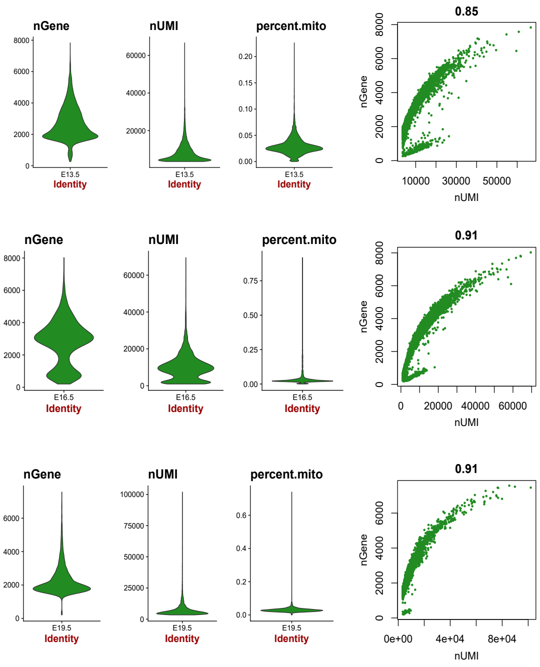

C

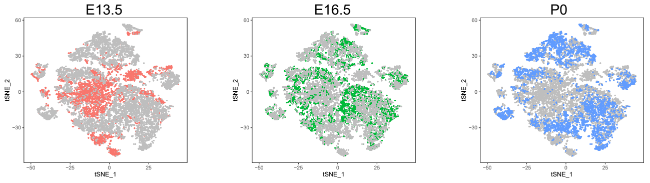

D

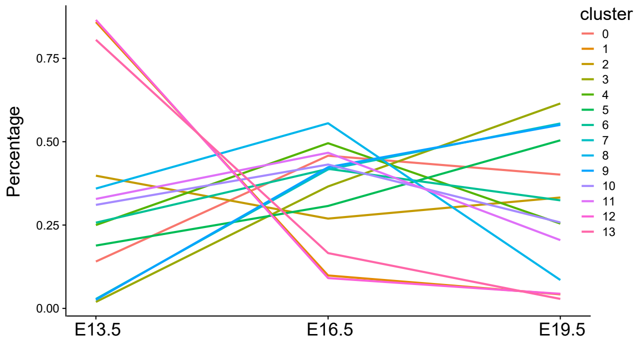

Figure S1: Quality control of single-cell data. (A) Single cell datasets quality metrics summary identified by CellRanger. (B) Violin plot displaying the number of genes (nGene), UMI (nUMI), and percentage of mitochondrial genes (percent.mito) detected in all single cells from three different datasets. The gene to UMI relationship for each dataset was also visualized. Generally, the more UMI captured, the more genes detected. (C) tSNE plot labeled by developmental timepoint. (D) Line-plot demonstrating the percentage of cells from different time-points in each cluster.

Supplementary Figure 2

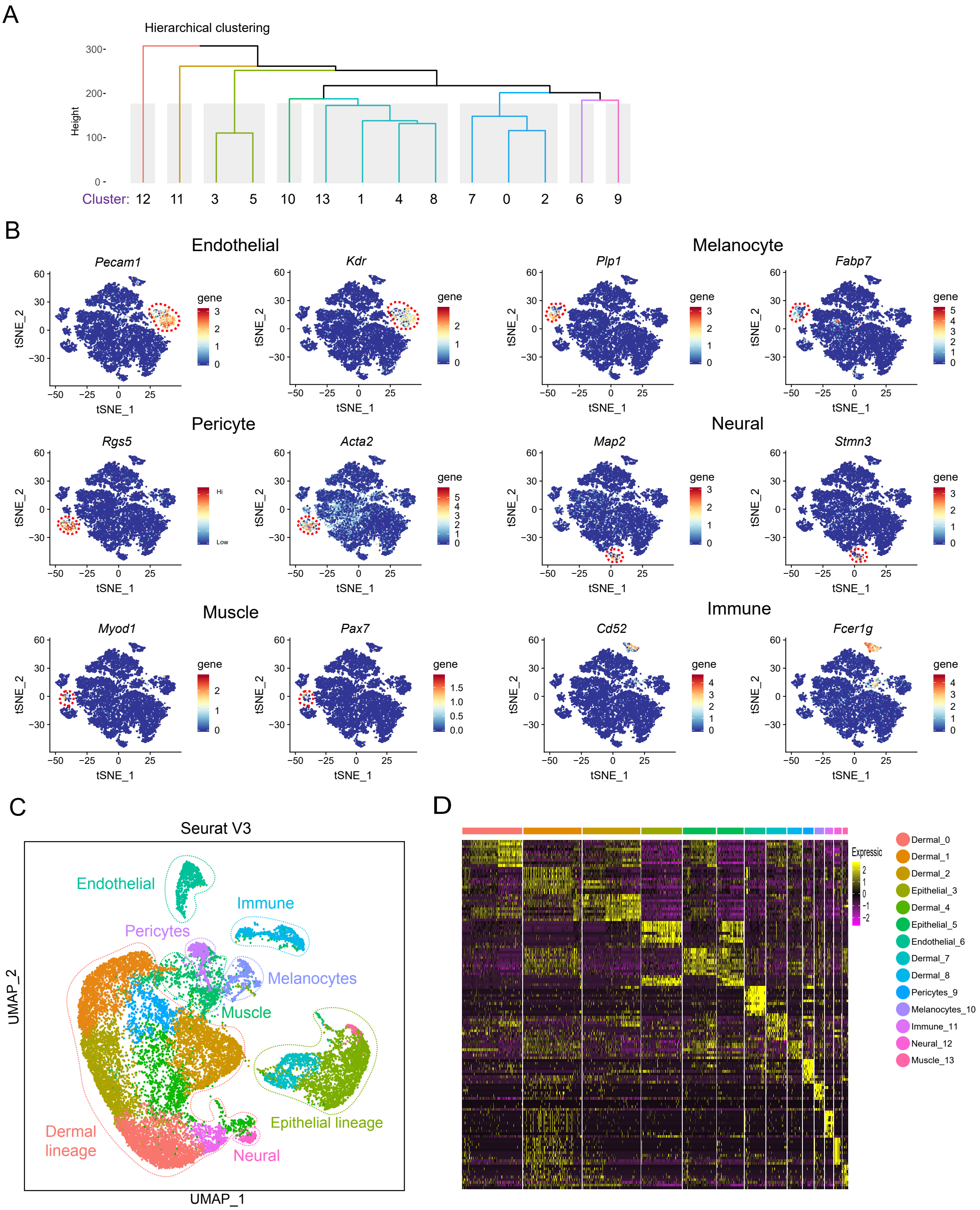

Figure S2: Characterization of major cell populations in the embryonic skin. (A) Hierarchical clustering of different cell clusters identified by tSNE. the cluster number was in accordance with Figure 1B. (B) Visualization of canonical maker gene expression in the tSNE plot of all single cells. Endothelial markers: *Pecam1*, *Kdr*; Melanocyte markers: *Plp1*, *Fabp7*; Pericyte markers: *Rgs5*, *Acta2*: Neural markers: *Map2*, *Stmn3*: Muscle markers: *Myod1*, *Pax7*, Immune markers: *Cd52*, *Fcer1g*. (C) Dimension reduction analysis using Seurat V3 pipeline. (D) Heatmap displaying top 10 signature gene expression in each cluster.

Supplementary Figure 3

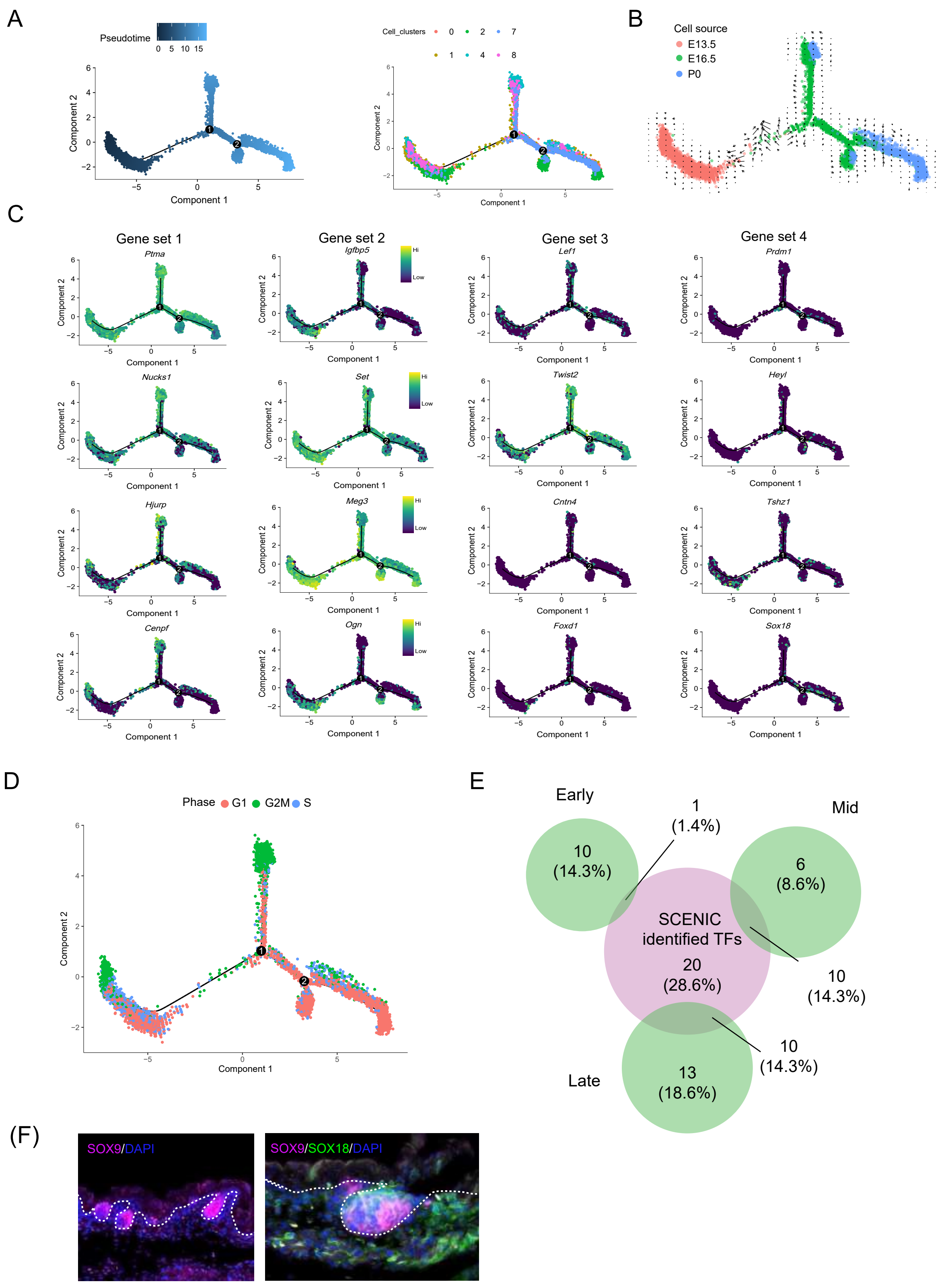

Figure S3: Visualization of key DC markers expression. (A) Pseudotime ordering analysis of all dermal populations, cells were labeled with pseudotime (left) and their corresponding cluster information (right), respectively. (B) RNA velocity vectors incorporated in the Monocle pseudotime trajectory. (C) Visulizing representative gene expression along pseudotime. (D) Visualizing cell cycle progression in the Monocle pseudotime trajectory. (E) Venn diagram showing overlapped transcriptional factors between this paper and Mok et al. Early, middle, and late represent different stages during hair follicle DC specification. (F) Immunofluorescence analysis of SOX9 and SOX18 expression in E16.5 skin tissues.

Supplementary Figure 4

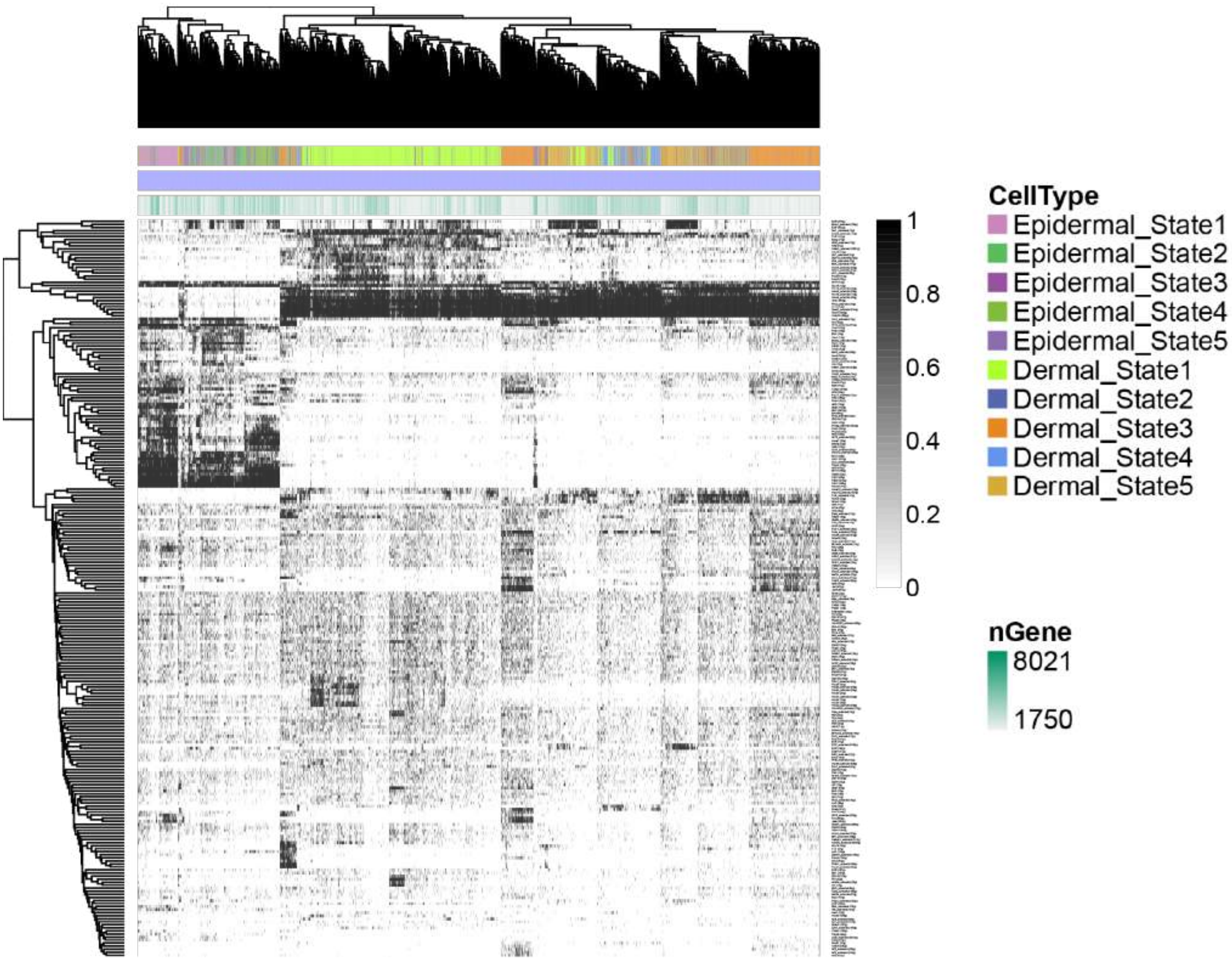

Figure S4: SCENIC binary regulon activity heatmap depicting all dermal and epidermal cell states-specific enriched regulons.

Supplementary Figure 5

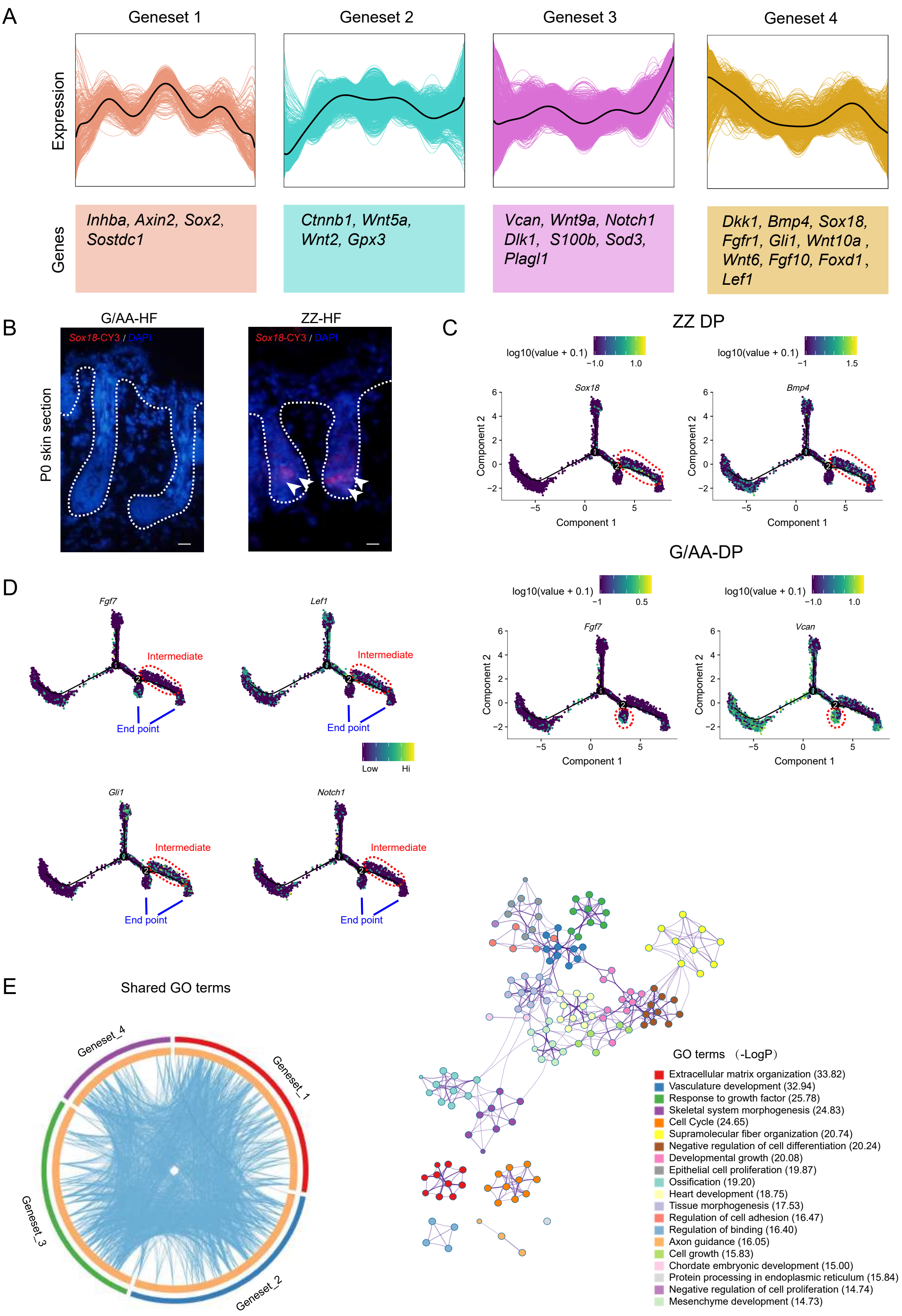

Figure S5: Investigating gene expression profile during AA/ZZ DP and G-DP fate commitment (A) Gene expression profile and representative genes during AA/ZZ DP and G-DP fate commitment. (B) mRNA ISH of *Sox18* in P0 skin tissue. (C) Representative AA/ZZ DP and G-DP marker expression projected into pseudotime trajectory. (D) Comparison of *Fgf7*, *Lef1*, *Gli1*, and *Notch1* expression in the pseudotime trajectory. The red dotted box depicts an intermediate stage, while the blue line indicates the endpoint. (E) Circos plot indicating the shared GO terms and GO interaction network constructed from different gene set enriched GO terms. Different colors in the network depict different GO terms.

Supplementary Figure 6

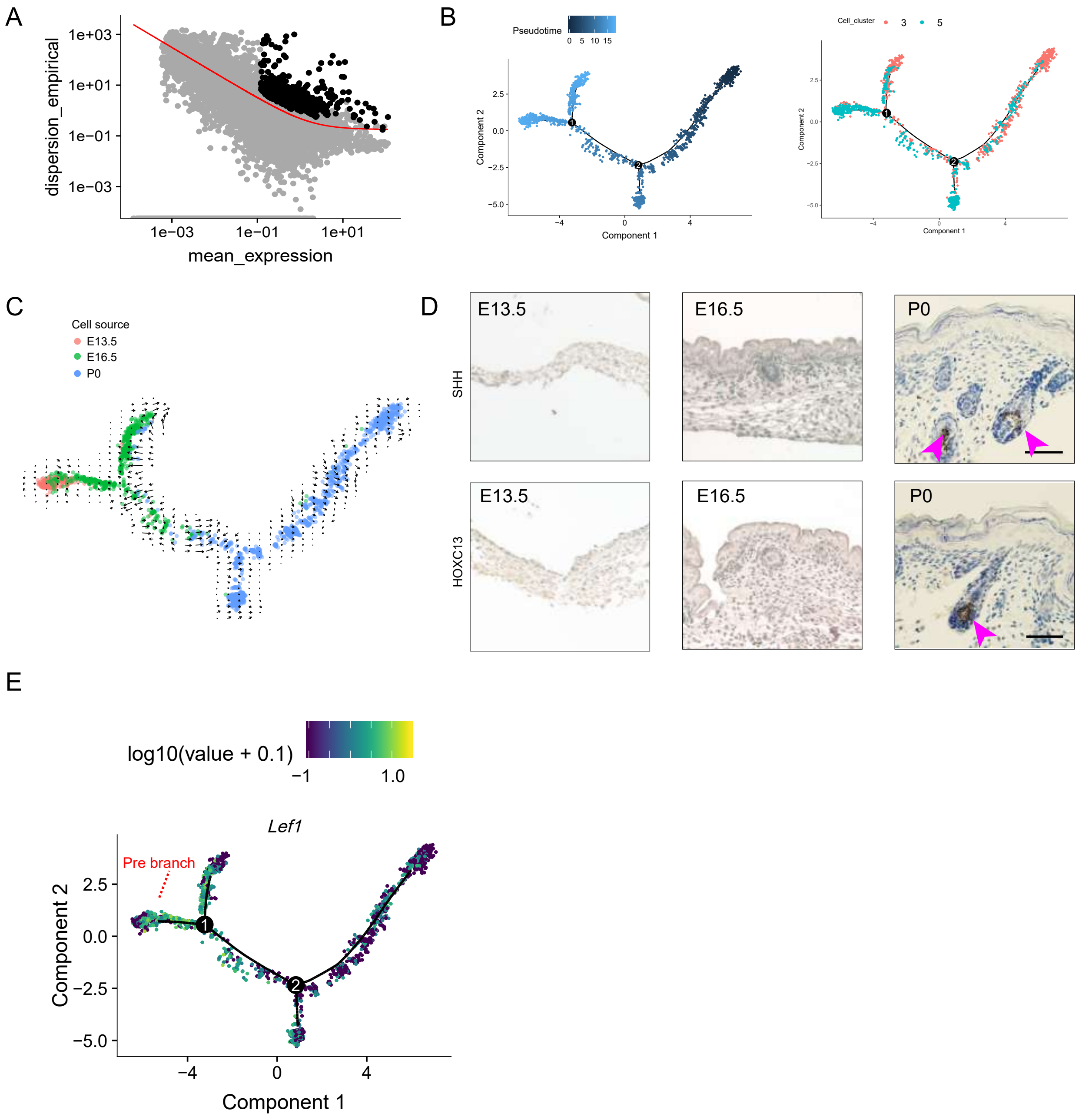

Figure S6: Interpreting IFE and matrix molecular signature (A) Scatter plot of mean expression against the empirical dispersion displaying the variable genes (black dots) used for Monocle pseudotime ordering. (B) Pseudotime ordering analysis of all epithelium populations, cells were labeled with pseudotime (left) and their corresponding cluster information (right), respectively. (C) RNA velocity vectors incorporated in the Monocle pseudotime trajectory. Cells were color-coded with their corresponding developmental timepoint. (D) Immunohistochemistry validation of SHH and HOXC13 expression in E13.5, E16.5 and P0 skin tissues. Scale bar, 50 µm. (F) Visualization of *Lef1* expression projected into pseudotime trajectory.

Supplementary Figure 7

A

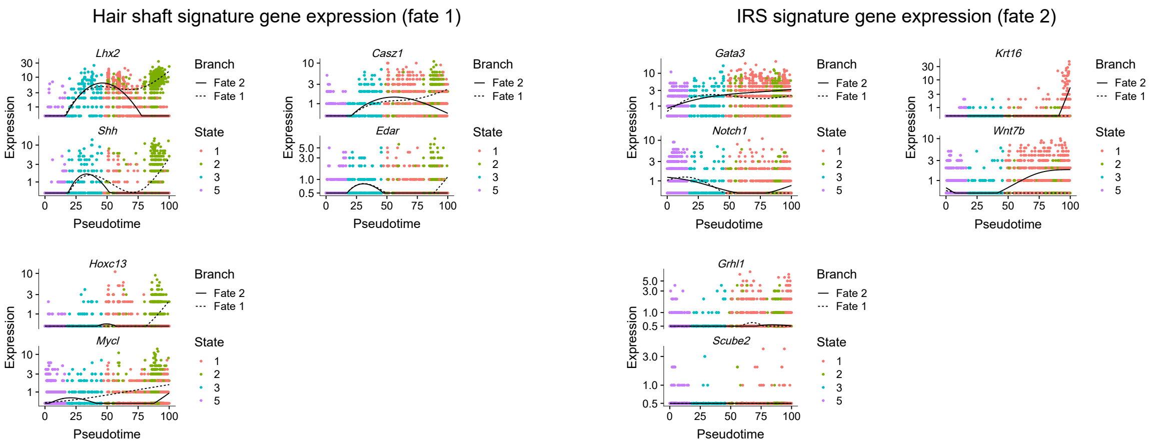

B

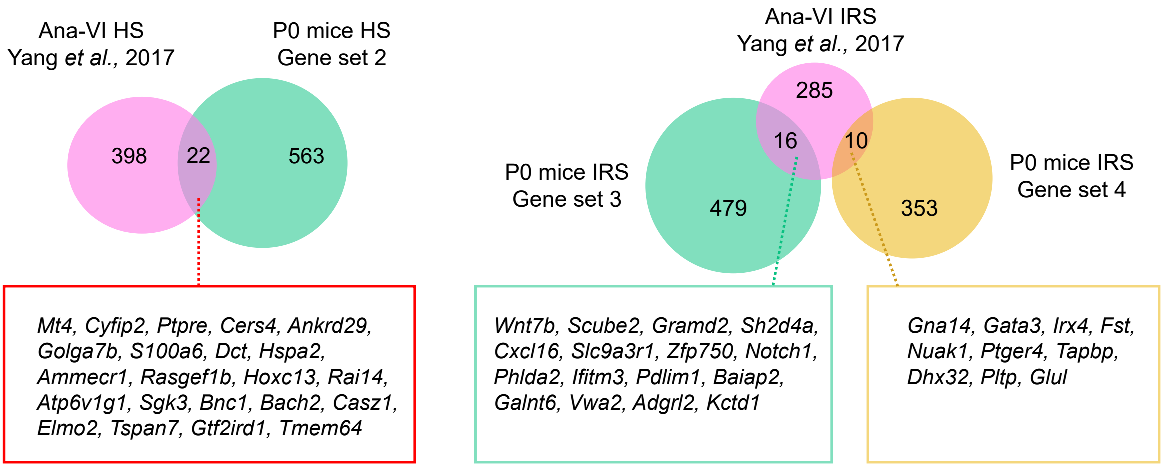

Figure S7: Dissecting hair shaft and IRS fate decisions. (A) Hair shaft and IRS signature gene expression along pseudotime. Cells were labeled with cell states with a solid line indicating fate 2 and a dotted line indicating fate 1. (B) Comparison of HS and IRS signature genes between Anagen VI from Yang et al., and our single cell analysis here. The overlapped genes were listed in the rectangular box.

Supplementary Tables:

Table S1. List of DEGs for tSNE identified 13 clusters. Corresponding to Figure 1B.

Table S2. List of branch-specific DEGs expression along pseudotime. Corresponding to Figure 2C.

Table S3. List of SCENIC enriched TFs and it's corresponding targets.

Table S4. Signature genes comparison P0 G-DP, AA/ZZ DP (this study) and AA/ZZ DP, ZZ DP, GAA DP, G-DP (Rezza et al.). Corresponding to Figure 3D.

Table S5. Signature genes comparison P0 hair shaft/IRS (this study) and anagen VI HS/IRS (Yang et al. 2017).
